# Multiple lineage-specific epigenetic landscapes at the antigen receptor loci

**DOI:** 10.26599/agr.2023.9340010

**Published:** 2023-08-24

**Authors:** Xiang Qiu, Guanxiang Liang, Weiqiang Zhou, Ranjan Sen, Michael L. Atchison

**Affiliations:** 1 Department of Biomedical Sciences, School of Veterinary Medicine, University of Pennsylvania, Philadelphia, Pennsylvania 19104, USA; 2 Laboratory of Molecular Biology and Immunology, National Institute on Aging, Baltimore, Maryland 21224, USA; 3 Department of Biostatistics, Johns Hopkins University Bloomberg School of Public Health, Baltimore, Maryland 21205, USA

**Keywords:** antigen receptors (AgRs), epigenetics, cell specificity, histone modifications, transcription

## Abstract

Antigen receptors (AgRs) expressed on B and T cells provide the adaptive immune system with ability to detect numerous foreign antigens. Epigenetic features of B cell receptor (BCR) and T cell receptor (TCR) genes were previously studied in lymphocytes, but little is known about their epigenetic features in other cells. Here, we explored histone modifications and transcription markers at the BCR and TCR loci in lymphocytes (pro-B, DP T cells, and mature CD4^+^ T cells), compared to embryonic stem (ES) cells and neurons. In B cells, the BCR loci exhibited active histone modifications and transcriptional markers indicative of active loci. Similar results were observed at the TCR loci in T cells. All loci were largely inactive in neurons. Surprisingly, in ES cells all AgR loci displayed a high degree of active histone modifications and markers of active transcription. Locations of these active histone modifications in ES cells were largely distinct from those in pro-B cells, and co-localized at numerous binding locations for transcription factors Oct4, Sox2, and Nanog. ES and pro-B cells also showed distinct binding patterns for the ubiquitous transcription factor YY1 and chromatin remodeler Brg1. On the contrary, there were many overlapping CCCTC-binding factor (CTCF) binding patterns when comparing ES cells, pro-B cells, and neurons. Our study identifies epigenetic features in ES cells and lymphocytes that may be related to ES cell pluripotency and lymphocyte tissue-specific activation at the AgR loci.

## 1 Introduction

The adaptive immune system can recognize various antigens through receptors expressed on B cells and T cells. The B cell receptor (BCR) consists of identical pairs of immunoglobulin heavy (Igh) and light (Igκ or Igλ) chain proteins. There are also multiple T cell receptor (TCR) loci (*Tcrα*, *Tcrδ*, *Tcrβ*, and *Tcrγ*) for similar pairs of proteins (αβ and γδ). Fully functioning antigen receptors are assembled by DNA rearrangements juxtaposing variable (V), diversity (D), and joining (J) gene segments, in a process called V(D)J recombination. BCR genes undergo rearrangements in the bone marrow at the pro-B and pre-B stages, and the TCR genes undergo rearrangement in the thymus in double negative (DN) and double positive (DP) T cells (Fig. S1 in the electronic supplementary material (ESM)) [1–4].

Histone post-translational modifications including acetylation, ubiquitylation, sumoylation, methylation, and phosphorylation generate a histone code that correlates with and may be causative of functional states within the genome [5–9]. The antigen receptor (AgR) loci span several hundred kilobases to several megabases, and undergo rearrangement and transcriptional activation tissue-specifically. Epigenetic modifications are believed to regulate these processes [4, 10–20].

The *Igκ* locus exhibits active histone modifications such as H3K4me1, H3K4me2, and H3K9ac in pro-B cells, similar to the *Igh* locus. The TCR loci are also enriched with histone modifications that are associated with chromatin conformational structure and V(D)J rearrangement [21–24]. In addition, non-coding RNA can function as direct and indirect modulators of epigenetic regulation [25, 26], and germline transcripts are expressed at pro-B and various T cell stages [19, 27–29]. Understanding histone modification differences in development is important as reduced TCR repertoire is responsible for impaired cellular immunity to inactivated severe acute respiratory syndrome coronavirus 2 (SARS-CoV-2) vaccine in older adults, and plasma cells that express various antibody isotypes are reduced in number during aging [30, 31].

Architectural protein CTCF, and many transcription factors are essential for lymphocyte development and are important regulators of the epigenetic landscape. Large-scale and shorter-range chromosomal loops are believed to be, at least partly controlled by the binding of cohesion and CTCF to loop anchor sites [28, 32–37]. Some of these loops define topologically associating domains (TADs). TAD loop anchors are believed to insulate sequences within TADs from interactions outside of the domain [32–34, 38–40]. Various other transcription factors bind to DNA sequences within TADs, to control the functional expression of encoded genes. Thus, binding of specific transcription factors to AgR loci is believed to control histone modifications and transcriptional activity [13, 19, 29, 41, 42]. In the immune system, these transcription factors include Pax5, EBF1, Ikaros, PU.1, YY1, E2A, Foxo1, Brg1, Id3, and TCF1 among others [43–59].

A variety of studies using primary mouse cells have presented ChIP-seq data for histone modifications at the BCR and TCR loci in pro-B or thymic T cells (Table S1 in the ESM), but very few comparisons have been made between them and other cell types. Here, we show data from multiple ChIP-seq studies presenting histone modifications and specific transcription factor binding at the BCR and TCR loci in mouse pro-B cells, DPT cells, mature CD4^+^ T cells, embryonic stem (ES) cells, and neurons. Our analyses show that histone modifications within the BCR and TCR loci in lymphocytes yield many histone modifications representative of active loci. These active epigenetic marks are largely absent in neurons. Strikingly however, we find that the AgR loci in ES cells are packaged with nucleosomes containing typically active histone modifications, and this feature may be at least partly the consequence of DNA binding by Oct4, Sox2, and Nanog, which dictates lineage-specific histone modification patterns distinct from those in the B cell lineage.

## 2 Results

### 2.1 Lineage-specific epigenetic signatures at BCR and TCR loci in ES cells, pro-B cells, DP T cells, mature CD4^+^ T cells, and neurons

Histone modifications provide an important measure of the structure and accessibility of genomic sequences. H3K4me1 is often associated with DNA hypomethylation, and general chromatin openness, H3K4me2 with tissue-specific regulation [60], H3K9ac with active promoters [61], and H3K27ac with active enhancers [62]. As the BCR and TCR loci alter their epigenetic states and accessibility to enable V(D)J recombination in the appropriate cell types, we evaluated epigenetic states of these loci in pro-B cells, DP T cells, mature CD4^+^ T cells, ES cells, and neurons (data sources and genomic coordinates are provided in Table S1 in the ESM).

### 2.2 Igκ

**Figure 1:**
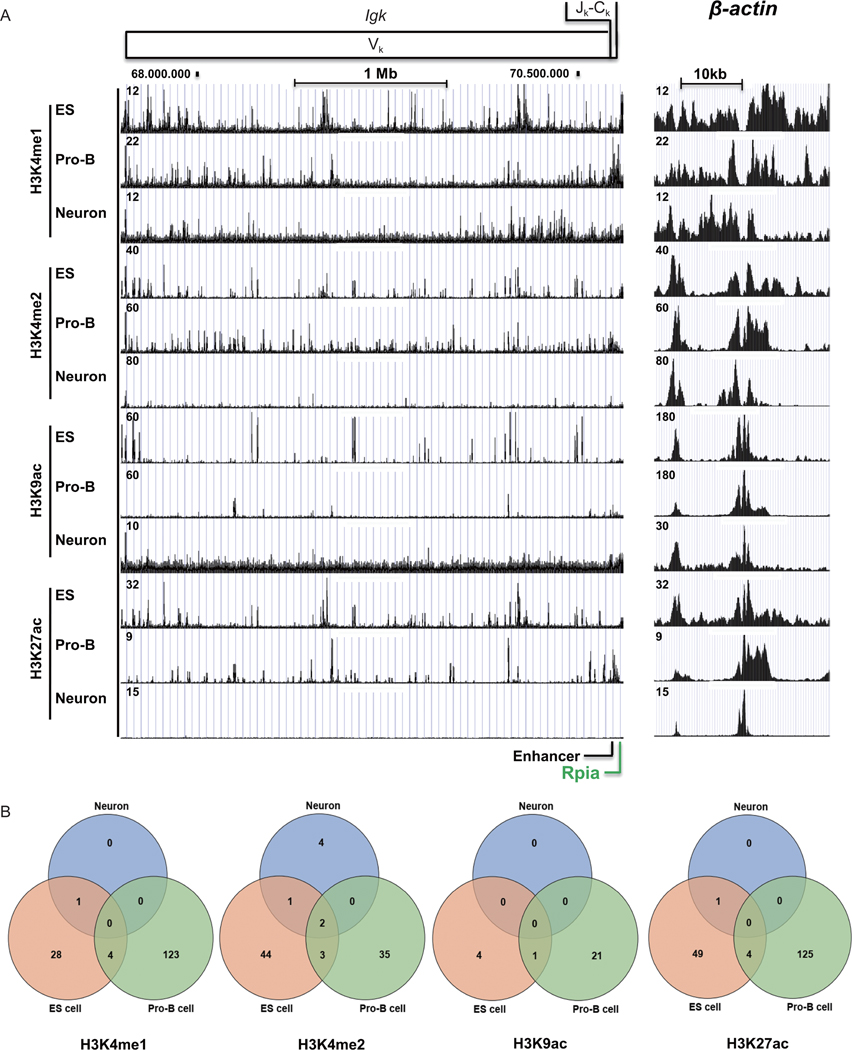
Histone modifications at the *Igκ* locus in ES cells, pro-B cells, and neurons show cell-specific patterns. (A) ChIP-seq data for H3K4me1, H3K4me2, H3K9ac, and H3K27ac are presented for the *Igκ* and *β-actin* loci. A map of the *Igκ* locus is shown in the top panel. The ribose-5-phosphate isomerase A gene (Rpia) flanks the locus on the 3’- and position of the *Ig* intron enhancer is indicated (bottom of panel A). Rpia is a non-*Ig* locus gene. Most peak patterns are distinct comparing the 3 cell types, though there is some degree of overlap within the 10kb Jκ-Cκ region. ChIP-seq data at the *β-actin* locus (right panels) (Chr5:142,891,416–142,920,715, mm10) was used as an internal control and for normalization between ES cells, pro-B cells, and neurons. (B) Venn diagrams of reproducible H3K4me1, H3K4me2, H3K9ac and H3K27ac peaks from ES cells, pro-B cells and neurons at the *Igκ* locus. We analyzed triple duplicates for each histone ChIP-seq data from ES cells, pro-B cells, and neurons. Numbers of peaks from each ChIP-seq dataset are shown in Table S2 in the ESM. Peaks were predicted by MACS2 with *q* < 0.05 [79]. The consensus peaks were identified by MSPC algorithm from triplicates [80].

The *Igκ* locus in mouse covers about 3.4 Mb. Recombination of V_κ-_J_κ_ occurs in pre-B cells, but the conformational structure of the locus is initiated in pro-B cells prior to rearrangement [15]. Therefore, we analyzed *Igκ* locus epigenetic features in pro-B cells, and compared this to features in ES cells and neurons. Signals over the β-actin gene were used to normalize signals between tracks representing various antibodies and cell types. H3K4me1 ChIP-seq data showed numerous peaks at the *Igκ* locus in pro-B cells, particularly within the distal and proximal regions, with some reduction in the central region of the locus (Fig. 1A, H3K4me1 tracks). Somewhat surprisingly, ES cells and neurons also showed numerous peaks as well, though most individual peaks did not overlap those in pro-B cells (Fig. 1A). MACS2 and MSPC algorithm analyses with triple duplicate ChIP-seq data sets (Table S2 in the ESM) enabled definition of peaks that were reproducibly observed. This showed that the peaks in neurons represented background signal, whereas 127 peaks in pro-B and 33 peaks in ES cells were reproducibly observed (Fig. 1B). Consistent with the visual pattern in Fig. 1A, there was a minimal overlap between the 127 peaks in pro-B cells with the peaks in ES cells (Fig. 1B, H3K4me1 Venn diagrams, 3.3% of pro-B peaks overlap peaks in ES cells). Similar results were observed with H3K4me2 ChIP-seq (Fig. 1A, H3K4me2 tracks). Whereas pro-B and ES cell peaks were consistently observed in multiple samples, these peaks did not appreciably overlap (Fig. 1B, H3K4me2 Venn diagram, only 14.3% of pro-B peaks overlapped ES cell peaks). The peaks in neurons again represented largely background signal. The results in pro-B cells and neurons are consistent with general tissue-specific patterns detected by H3K4me2, but are surprising in ES cells.

A number of peaks in pro-B cells were detected by H3K9ac ChIP-seq (Fig. 1A, H3K9ac tracks), which generally detects active promoters. Again, there were a surprisingly high number of peaks in ES cells, but very few of these were consistent in multiple data sets, and only 1 of these peaks overlapped the peaks in pro-B cells (Fig. 1B, H3K9ac Venn diagram, 4.8% of pro-B peaks overlap ES cell peaks). There were no reproducible peaks in neurons representing background signals. In addition, H3K27ac peaks, which generally mark active enhancers, were prominent in both pro-B cells and ES cells (Fig. 1A) but very few of these peaks overlapped (Fig. 1B, H3K27ac Venn diagram, 3.7% of pro-B peaks overlapped ES cell peaks). Neurons showed essentially no reproducible peaks (Figs. 1A and 1B).

**Figure 2:**
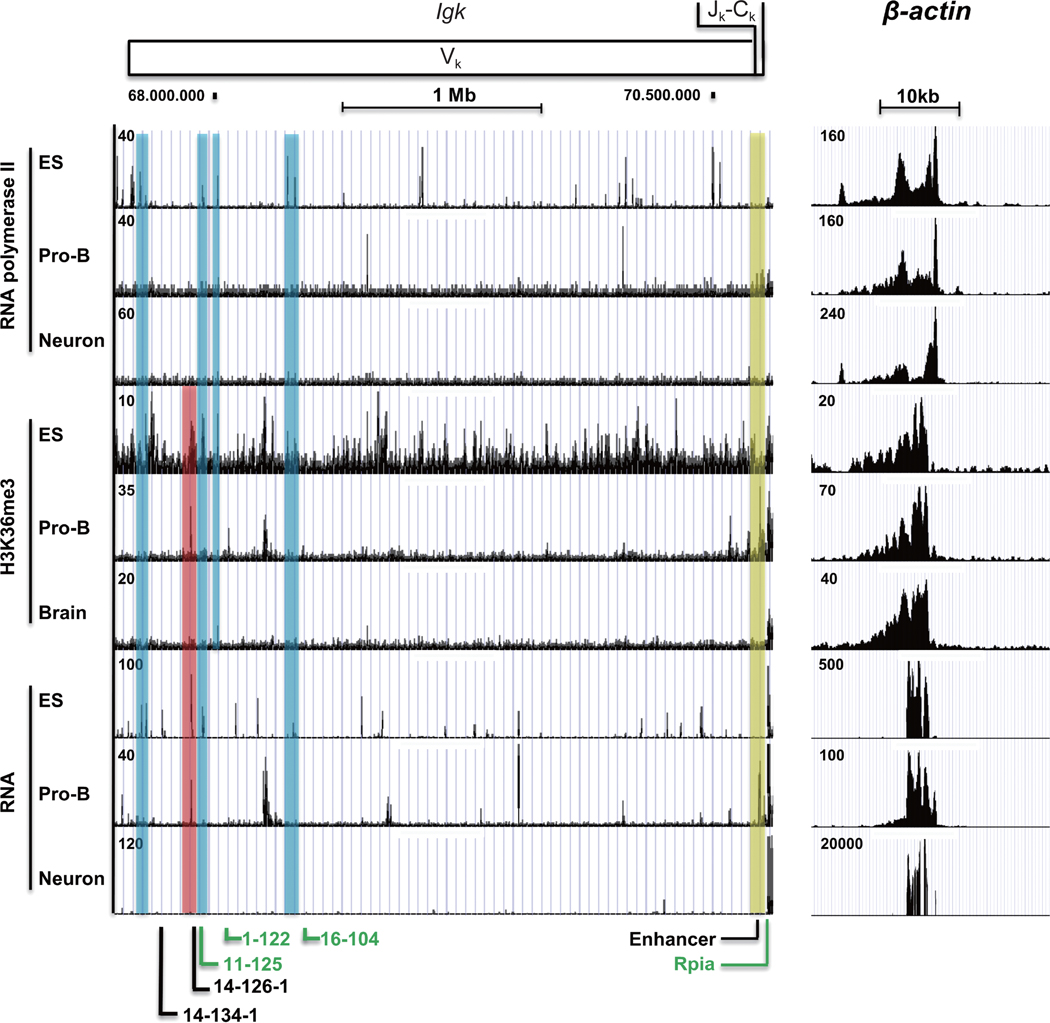
Transcription markers at the *Igκ* locus in ES cells, pro-B cells, and neurons. ChIP-seq data for RNA polymerase II and H3K36me3, and RNA-seq data are shown for the *Igκ* and *β*-actin loci. The *Igκ* locus shows surprising activity in ES cells with all three markers (RNA pol II, H3K36me3, and RNA transcripts). Blue, yellow and red shading indicate peaks only in ES cells, pro-B cells or both, respectively. Functional and pseudo gene segments at the *Igκ* locus are labeled in green and black color, respectively. ChIP-seq data at the *β*-actin locus (Chr5:142,891,416–142,920,715, mm10) is used as an internal control and for normalization between ES cells, pro-B cells, and neurons. Rpia is a non-*Ig* locus gene.

To more directly evaluate transcriptional activity of the *Igκ* locus, we evaluated ChIP-Seq data using transcription markers including RNA polymerase II and H3K36me3 (which is associated with transcriptional elongation) [63, 64], as well as RNA transcripts from RNA-Seq data. RNA pol II ChIP-Seq showed two prominent peaks over the *Igκ* locus in pro-B cells, with an additional cluster in the J_κ_-C_κ_ region (Fig. 2). The low number of RNA pol II peaks is not surprising, as the locus is not yet undergoing V_κ_-J_κ_ rearrangement at this stage. Surprisingly, there were multiple RNA pol II peaks in ES cells, distinct from those in pro-B cells, and no strong peaks were observed in neurons (Fig. 2). H3K36me3 peaks did not correspond with the RNA pol II peaks in pro-B cells, except within the J_κ_-C_κ_ region (Fig. 2, yellow shading). In ES cells, the *Ig* locus displayed numerous low level H3K36 peaks with little overlap with those in pro-B cells (Fig. 2). Finally, RNA transcript data yielded 8–10 strong peaks spanning the *Ig* locus in pro-B cells. Even more peaks were observed in ES cells, all of which were distinct from peaks in pro-B cells except for one peak (Fig. 2, red shade). The H3K36me3 peaks in each cell type in some cases matched the corresponding RNA transcript data, though the peaks for RNA pol II were often distinct (Fig. 2), indicating that the *Igκ* locus is differentially transcriptionally active in both pro-B and ES cells, but not strongly transcriptionally active in neurons.

Blue, yellow, and red shading refer to peaks in only ES cells, pro-B cells, and both, respectively. Transcript reads across the *Ig* locus are compared to reads across the entire genome in Table S4 in the ESM.

### 2.3 Igh

**Figure 3:**
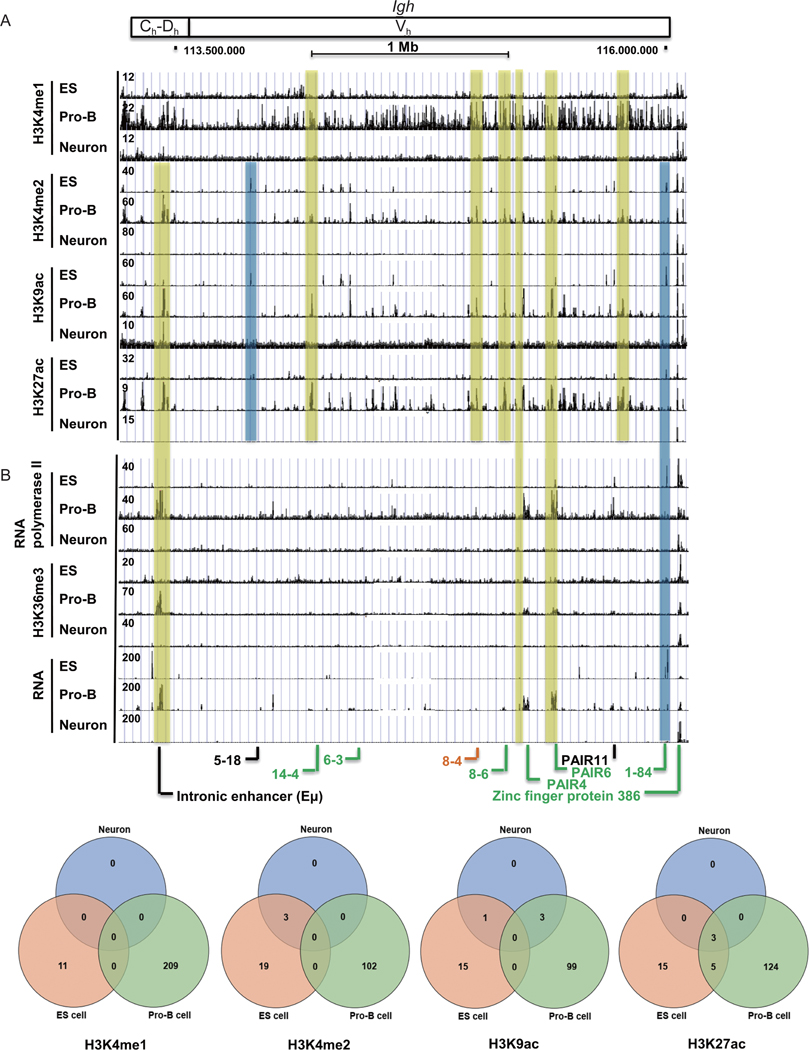
Histone modifications and transcription markers at the *Igh* locus in ES cells, pro-B cells, and neurons. ChIP-seq data for histone modifications (A) and transcription markers (B) are shown for the *Igh* locus. Blue and yellow shading refer to peaks in only ES cells or only pro-B cells, respectively. Functional gene segments, pseudogenes and open reading frames at the *IgH* locus are labeled in green, black and orange color, respectively. Zinc finger protein 386 is a non-*Ig* locus gene. (C) Venn diagrams of reproducible H3K4me1, H3K4me2, H3K9ac, and H3K27ac peaks from ES cells, pro-B cells and neurons at the *Igh* locus. We analyzed triple duplicates from multiple histone ChIP-seq data from ES cells, pro-B cells, and neurons. Numbers of peaks from each ChIP-seq are shown in Table S3 in the ESM. Peaks were predicted by MACS2 with *q* < 0.05 [79]. The consensus peaks were identified by MSPC algorithm from triplicates [80].

The *Igh* locus spans 2.4 Mb and contains constant (C_h_), joining (J_h_), diversity (D_h_), and variable (V_h_) gene segments. Histone modifications at the *Igh* locus in pro-B cells displayed lineage-specific patterns with a fairly tight association of peaks for H3K4me2, H3K9ac, and H3K27ac (Fig. 3A, see pro-B tracks, yellow shading). The H3K4me1 pattern was more diffuse and included many of the same peaks, but it additionally showed a broad assortment of fairly low-level peaks (Fig. 3A), yet many of these peaks were consistently observed in multiple samples (Fig. 3C). Similar to the *Igκ* locus, a number of peaks for each histone modification were present at the *Igh* locus in ES cells, but these peaks were fewer in number compared to pro-B peaks (Fig. 3A, comparing ES to pro-B tracks, and Fig. 3C; Table S3 in the ESM). A small percentage (6.1%) of H3K27ac peaks overlapped between ES cell and pro-B peaks (Fig. 3A, red and blue shades, Fig. 3C). Essentially no reproducible peaks were observed in neurons (Fig. 3C). In summary, MACS2 and MSPC algorithm analyses showed that none of the reproducible pro-B cell peaks for the histone modifications H3K4me1, H3K4me2, or H3K9ac overlapped those in ES cells, and only 6.1% of pro-B cell H3K27ac peaks overlapped ES cell peaks (Fig. 3C).

RNA polymerase II, H3K36me3, and RNA transcripts, were observed as co-localizing peaks in pro-B cells within the J_h_-C_h_ region, as well as within the distal V_h_ locus (Fig. 3B, yellow shade) consistent with active V(D)J rearrangement in pro-B cells [29, 47, 56]. Several of these peaks correspond to PAIR sequence transcripts or to non-coding transcripts across the Eμ enhancer (Fig. 3B, yellow shade). Some transcription markers were present in ES cells (Fig. 3B, blue shading), but very few peaks overlapped the pro-B peaks (Fig. 3B, blue shade showing ES only peaks). Very low signals were consistently observed in multiple data sets in neurons (Fig. 3B). Unlike the *Igκ* locus, RNA polymerase II, H3K36me3, and RNA showed considerable co-localization of peaks in pro-B cells (Fig. 3B, yellow shade). Overall, active histone marks and transcription markers were enriched at the *Igh* locus in pro-B cells compared to the other cell types. Transcript reads across the *Ig* locus are compared to reads across the entire genome in Table S2 in the ESM.

### 2.4 Tcrα/δ

**Figure 4:**
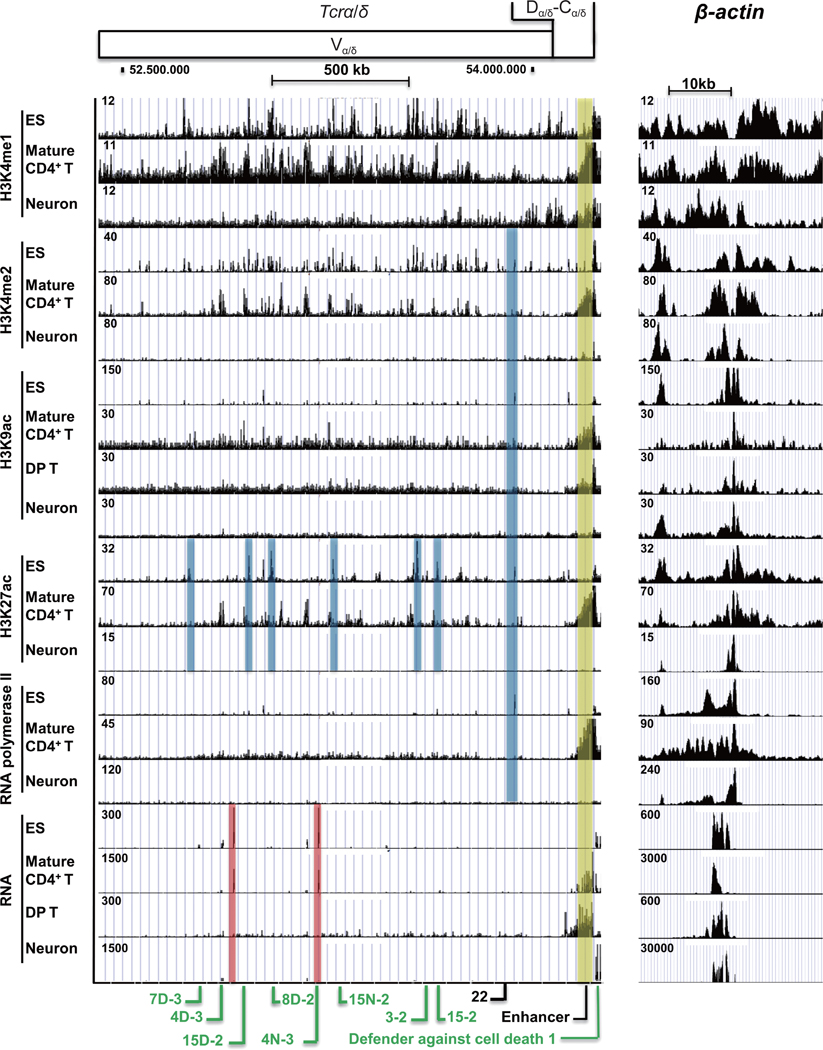
Histone modifications and transcription markers at the *Tcrα/δ* locus in ES cells, DP T cells, mature CD4+ T cells, and neurons. ChIP-seq and RNA-seq data for histone modifications and transcription markers are shown for the *Tcrα/δ* and *β*-actin loci. Blue, yellow and red rectangles refer to peaks in only ES cells, mature CD4+ T cells, or both, respectively. Functional and pseudo gene segments at the *Tcrα/δ* locus are labeled in green and black colors, respectively. Defender against cell death 1 is a non-TCR gene. ChIP-seq data at the *β*-actin locus (right panel) (Chr5:142,891,416–142,920,715, mm10) is used as an internal control and for normalization between ES cells, mature CD4+ T cells, and neurons.

The *Tcrα/δ* locus is located on chromosome 14, spans 2.1 Mb, and comprises both the *Tcrα* and *Tcrδ* loci. DP T cells represent an early stage of T cell development just subsequent to TCR V(D)J rearrangement. However, given the limited available ChIP-Seq data from DP T cells, we also analyzed data from mature CD4^+^ T cells for the TCR loci. The *Tcrα/δ* loci in mature CD4^+^ T cells showed many low amplitude H3K4me1 peaks suggesting a high degree of openness. Numerous different peaks were also observed in ES cells (Fig. 4). Neurons showed far fewer peaks with greatest occurrence in the D_α/δ_-C_α/δ_ region (Fig. 4). H3K4me2 and H3K27ac peaks were prominent in both mature CD4+ T cells and ES cells, but were largely absent in neurons (Fig. 4). A number of peaks were predominantly in ES cells (blue shading) or mature CD4^+^ T cells (yellow shading). Fewer H3K9ac peaks were observed in both ES cells, DP T cells and mature CD4^+^ T cells (Fig. 4). RNA polymerase II bound in the 3’ portion of the *Tcrα/δ* locus in mature CD4^+^ T cells, with scattered peaks throughout the locus (Fig. 4). These RNA polymerase II peaks were fairly low in mature CD4+ T cells, but were absent in neurons. Prominent peaks were observed at the 3’ D_α/δ_-C_α/δ_ region. Several RNA transcript peaks were identified that matched in ES cells and mature CD4^+^ T cells (Fig. 4, red shade). Numerous non-coding RNA transcripts were derived from the 3’ enhancer in mature CD4^+^ and DP T cells. These data indicate that similar to the *Ig* locus, the *Tcrα/δ* loci are active in the appropriate cell type (DP T and CD4^+^ T cells), but also showed some activity in ES cells, while neurons showed low accessibility and low transcription (Fig. 4).

### 2.5 Tcrβ

**Figure 5:**
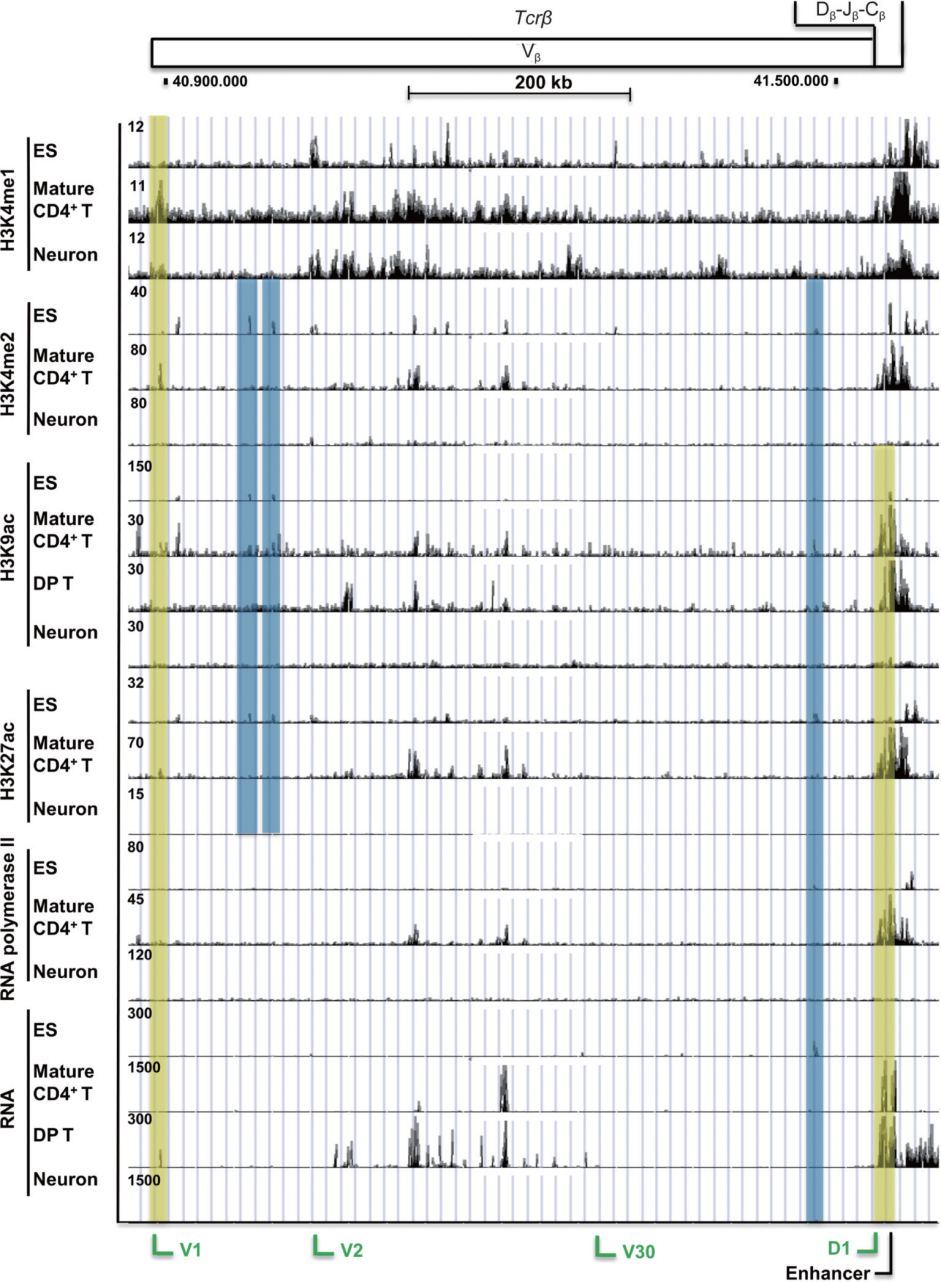
Histone modifications and transcription markers at the *Tcrβ* locus in ES cells, DP T cells, and mature CD4^+^ T cells, and neurons. ChIP-seq and RNA-seq data for histone modifications and transcription markers are shown for the *Tcrβ* locus. Blue and yellow rectangles refer to peaks in only ES cells or mature CD4^+^ T cells, respectively. Functional gene segments at the *Tcrβ* locus are labeled in green or black colors at the bottom.

The *Tcrβ* locus is located on chromosome 6, and covers about 700 kb. The locus shows diffuse openness (H3K4me1) in mature CD4^+^ T cells, ES cells, and surprisingly within neurons, though the patterns of peaks are largely distinct and peak amplitudes are low (Fig. 5). The tissue-specific peaks (H3K4me2) showed some degree of overlap between mature CD4^+^ T cells and ES cells, but in contrast to results with H3K4me1, there are very few peaks in neurons (Fig. 5). H3K9ac and H3K27ac peaks were largely specific to mature CD4^+^ T cells, as were the peaks for RNA pol II and RNA transcripts (Fig. 5). Similar to other antigen receptor loci, the enhancer at the *Tcrβ* locus in mature CD4^+^ and DP T cells is enriched with non-coding RNA transcripts. Overall, there was a good correlation of RNA polymerase II peaks and RNA transcripts at the *Tcrβ* locus in mature CD4^+^ T cells. Peaks present in ES cells only are indicated by blue shading, and those in mature CD4^+^ T cells alone by yellow shading.

Taken together, BCR and TRC loci in ES cells display a surprising degree of active histone modifications and transcription markers, with profiles that are distinct from lymphocytes. In most cases, the loci in neurons are essentially inactive.

### 2.6 ES cell-specific transcription factor enrichment atthe BCR and TCR loci

The high degree of active histone marks, and transcription markers in ES cells was surprising. We extended our analyses to ES cell-specific transcription factors Sox2, Oct4, and Nanog to explore if these factors might cause these histone modifications. These transcription factors are essential for maintaining the pluripotent activity and function of ES cells [65–68]. Therefore, we sought to determine if there was a correlation between these transcription factors and histone modifications at the AgR loci in ES cells.

### 2.7 Igκ

**Figure 6:**
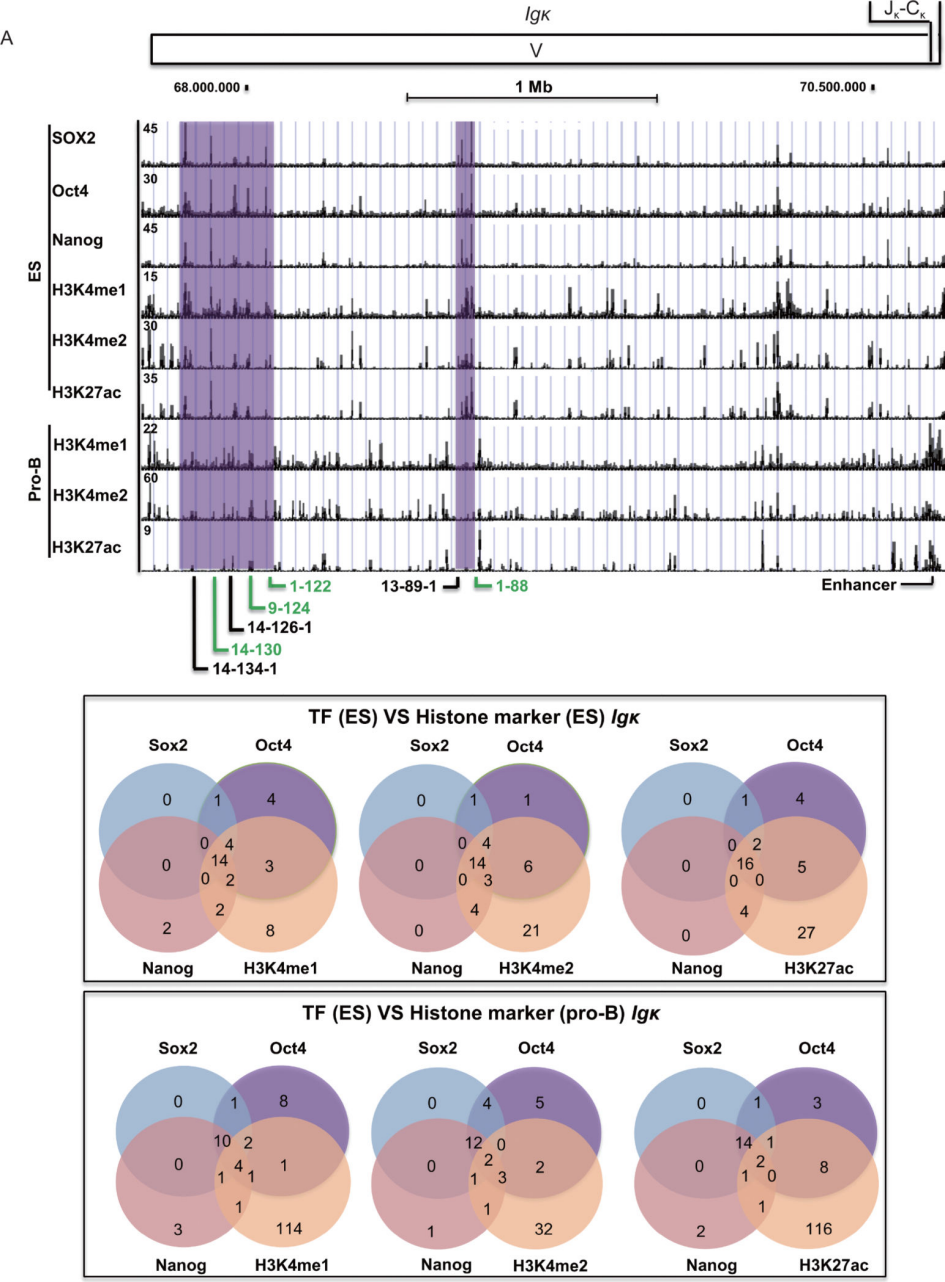
Correlation of transcription factor binding profiles and histone modifications at the *Igκ* locus in ES cells and pro-B cells. (A) Sox2, Oct4, and Nanog transcription factor binding profiles are compared to histone modifications at the *Igκ* locus in ES cells and pro-B cells. Purple shading refers to overlapping peaks for transcription factor binding and histone modifications in ES cells, but not in pro-B cells. Functional gene segments and pseudogenes are labeled in green and black colors, respectively. (B) Venn diagrams show the overlaps between reproducible Sox2, Oct4, and Nanog peaks, with reproducible H3K4me1, H3K4me2, or H3K27ac peaks. The top panel compares the overlap of transcription factor peaks and histone peaks in ES cells, and the bottom panel shows the positions of transcription factor peaks in ES cells, to the histone peaks observed in pro-B cells. We analyzed triple duplicates for each histone ChIP-seq data from ES cells, pro-B cells, and neurons. Numbers of peaks from each ChIP-seq dataset are shown in Table S2. Peaks were predicted by MACS2 with *q* < 0.05 [79]. The consensus peaks were identified by MSPC algorithm from triplicates [80].

At the *Igκ* locus, there was a strong co-localization of Sox2, Oct4, and Nanog peaks to 14 distinct sites in ES cells (Fig. 6A, purple shade; Fig. 6B, top panel). Overall, 100% of Sox2, 75% of Oct4, and 83% of Nanog sites overlapped with one or more of the other transcription factors (Fig. 6B). There was also a high correlation of many of the Sox2, Oct4 and Nanog peaks with peaks for H3K4me1, H3K4me2, and H3K27ac (Fig. 6B, top panel). 76% of H3K4me1, 60% of H3K4me3, and 50% of H3K27ac peaks overlapped with Sox2, Oct4, or Nanog peaks (Fig. 6B, top panel). Many of the Sox2, Oct4 and Nanog peaks localized to *Igκ* V genes (Fig. 6A, purple shade, bottom panel). However, the positions of ES cell peaks for Sox2, Oct4, Nanog, did not correspond well with the locations of histone modification peaks in pro-B cells where only 4%, 22%, and 5% of H3K4me1, H3K4me2, and H3K27ac peaks in pro-B cells overlapped with the corresponding positions of Sox2, Oct4, and Nanog peaks in ES cells (Fig. 6A, purple shade; Fig. 6B bottom panel). Thus, distinct transcription factors appear to control histone modifications in ES cells compared to pro-B cells.

### 2.8 Igh

**Figure 7:**
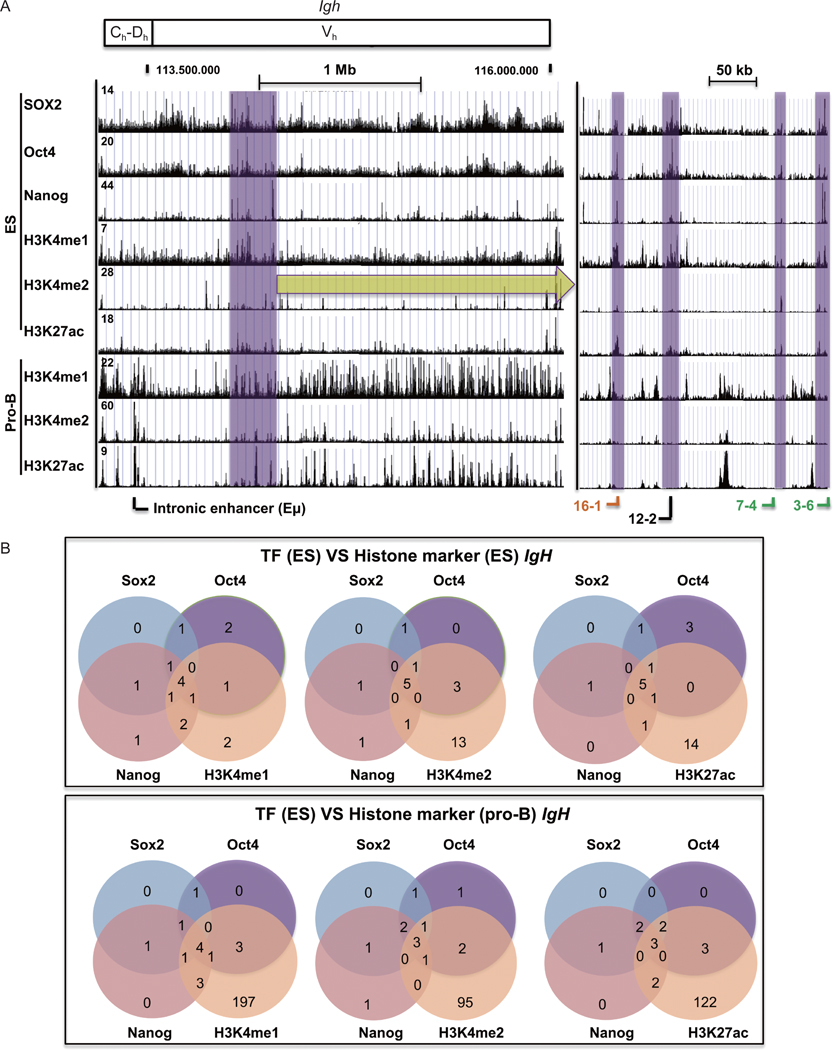
Correlation of transcription factor binding profiles and histone modifications at the *Igh* locus in ES cells and pro-B cells. (A) Sox2, Oct4, and Nanog transcription factor binding profiles are compared to histone modifications at the *Igh* locus in ES cells and pro-B cells. Purple shading refers to overlapping peaks for transcription factor binding and histone modifications in ES cells, but not in pro-B cells. The purple shaded region is exapaned in the right panel indicated by the yellow arrow. Functional gene segments, pseudogenes, and an open reading frames are labeled in green, black, and orange colors, respectively. (B) Venn diagrams show the reproducible overlaps between Sox2, Oct4, and Nanog peaks, with reproducible H3K4me1, H3K4me2, or H3K27ac peaks. The top panel compares the overlap of reproducible transcription factor peaks and histone peaks in ES cells, and the bottom panel shows the positions of transcription factor peaks in ES cells, to the histone peaks observed in pro-B cells. We analyzed triple duplicates for each histone ChIP-seq data from ES cells, pro-B cells, and neurons. Numbers of peaks from each ChIP-seq dataset are shown in Table S3 in the ESM. Peaks were predicted by MACS2 with *q* < 0.05 [79]. The consensus peaks were identified by MSPC algorithm from triplicates [80].

Compared with the *Igκ* locus, fewer prominent Sox2 and Oct4 peaks were observed at the *Igh* locus in ES cells, yielding a more diffuse binding pattern (Fig. 7A). MACS2 and MSPC algorithm analyses showed that reproducible Sox2, Oct4, and Nanog peaks in ES cells overlapped with one another (100% of Sox2, 75% of Oct4, and 91% of Nanog peaks overlapped with at least one of the other factors) (Fig. 7B, top panel). Some of the peaks that were observed in ES cells among the three transcription factors are shown in expanded scale in Fig. 7A, right panel. Interestingly, these peaks correspond to V_h_ genes (Fig. 7A). We found 81.8% of H3K4me1, 43.5% of H3K4me2, and 36.4% of H3K27ac peaks in ES cells overlapped with the three transcription factor peaks in ES cells (Fig. 7B, top panel). On the contrary, only 5%–7% of Sox2, Oct4, and Nanog sites in ES cells overlapped with the locations of H3K4me1, H3K4me2, and H3K27ac sites in pro-B cells (Fig. 7B, bottom panel).

### 2.9 Distinct YY1 and Brg1 binding profiles between ES cells and pro-B cells at the *Igκ* and *Igh* loci

**Figure 8:**
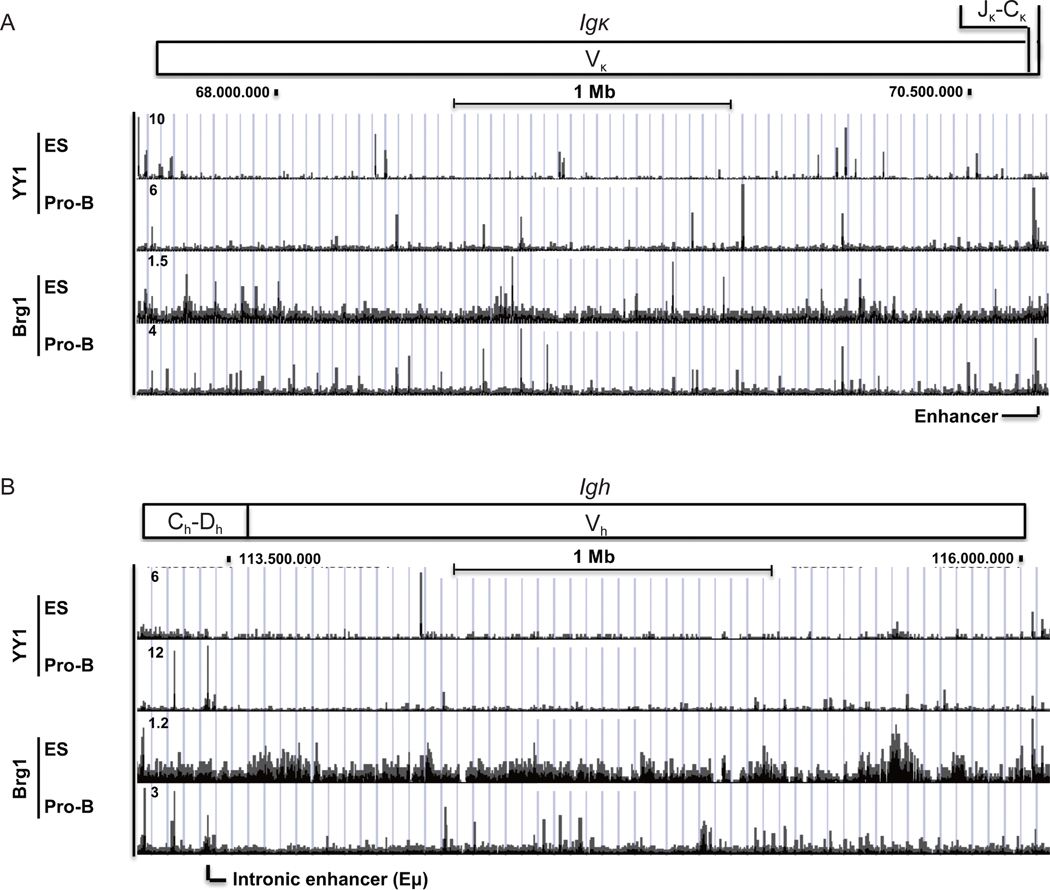
8 YY1 and Brg1 binding profiles and histone modifications at the *Igκ* and *Igh* loci in ES cells and pro-B cells. ChIP-seq data for YY1 and Brg1 are shown for *Igκ* (A) and *Igh* (B) loci in ES cells and pro-B cells.

It has been argued that tissue-specific transcription factors control the epigenetic landscape enabling ubiquitous factors such as YY1 to gain access to DNA to generate DNA structures (such as DNA loops) needed for gene regulation [69]. If true, one would anticipate that DNA binding of ubiquitous factors to the *Ig* loci would be different in pro-B and ES cells due to the distinct lineage-specific transcription factors expressed in these cell types. Therefore, we extended our analyses at the *Igκ* and *Igh* loci in pro-B and ES cells to conserved ubiquitous transcription factor YY1 and the chromatin remodeler Brg1, a component of the Swi/Snf complex expressed in both cell types. The *Igκ* locus displayed distinct binding patterns for YY1 and Brg1 comparing ES cells to pro-B cells (Fig. 8A), yielding little overlap in peaks in the two cell types. Similar results were observed at the *Igh* locus (Fig. 8B). Taken together, both the *Igκ* and *Igh* loci showed distinct YY1 and Brg1 binding profiles when comparing ES cells and pro-B cells, consistent with the model whereby their localization is dependent upon tissue-specific transcription factors.

### 2.10 Common binding patterns for CTCF

**Figure 9:**
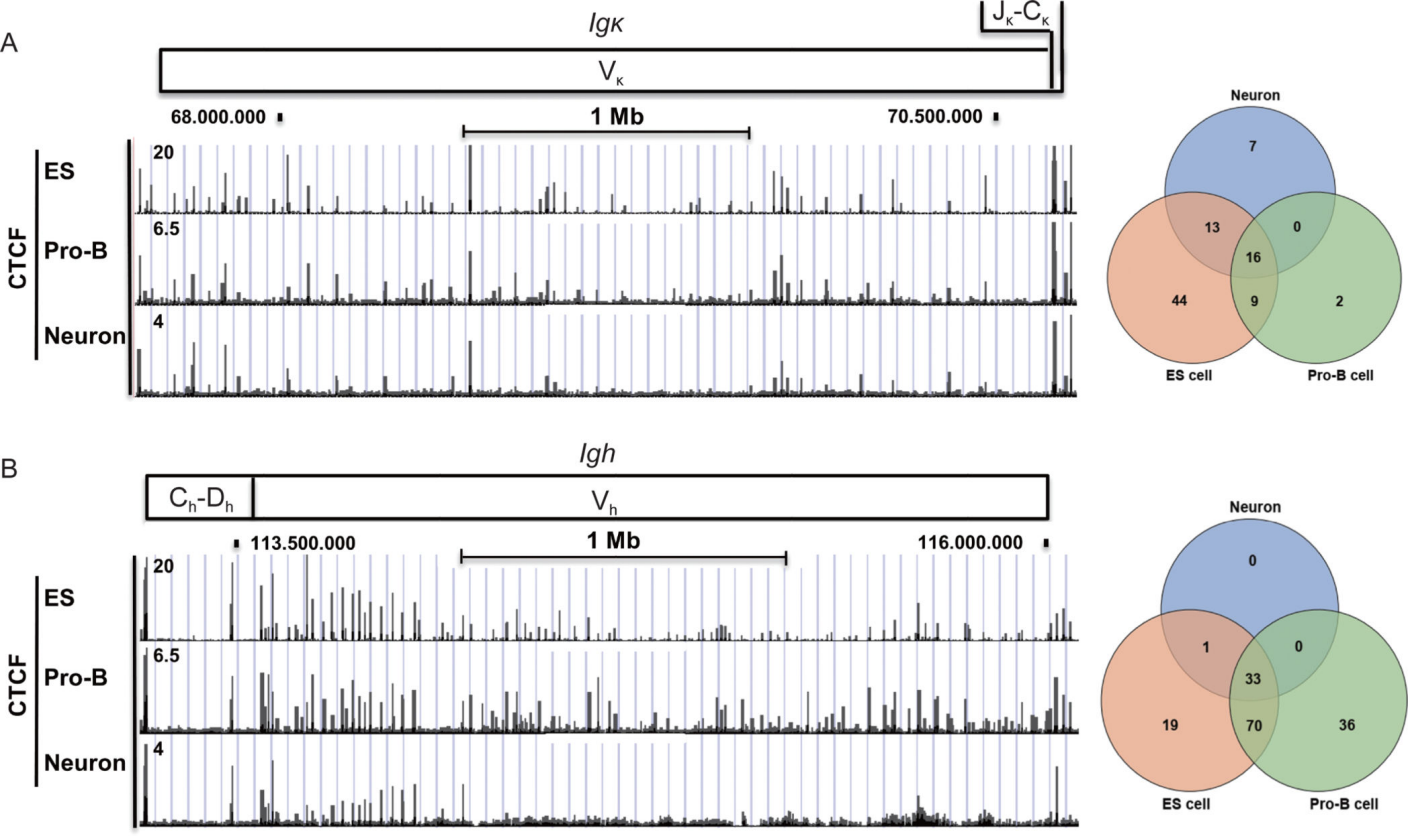
Conserved CTCF binding profiles at the antigen receptor loci among ES cells, pro-B cells, and neurons. ChIP-seq data for CTCF are presented for the *Igκ* (A) and *Igh* (B) loci. A map of each antigen receptor locus is shown in the top panel of each figure. Overlaps of reproducible CTCF peaks in ES cells, pro-B cells, and neurons are shown in the Venn diagrams in the right panels. We analyzed triple duplicates for CTCF ChIP-seq datasets from ES cells, pro-B cells, and neurons. Numbers of peaks from each ChIP-seq were shown in Tables S2 and S3 in the ESM. Peaks were predicted by MACS2 with *q* < 0.05 [79]. The consensus peaks were identified by MSPC algorithm from triplicates [80].

Our analyses unveiled lineage-specific epigenetic features at BCR and TCR loci, as well as correlations between specific transcription factors and lineage-specific epigenetic features in ES cells. We also sought protein binding patterns that might be common between multiple cell types. Indeed, we found CTCF ChIP-Seq profiles are similar at the *Igκ* and *Igh* loci in pro-B cells, ES cells, and neurons indicating that not all transcription factor binding profiles are distinct at the BCR loci in various cell types (Figs. 9A and 9B). For instance, 42% of all CTCF peaks overlapped at the *Ig* locus in pro-B cells, neurons, and ES cells, while 65% overlapped at the Igh locus (Figs. 9A and 9B). At the *Ig* locus in pro-B cells, 93% of reproducible CTCF peaks overlapped those in ES cells, and 59% in neurons (Fig. 9A). Loguercio et al. [70] also observed similar CTCF patterns at the *Igκ* locus comparing pro-B and ES cells, but considerable differences were observed when comparing pre-B patterns to ES cells at the *Igκ* locus presumably due to activation of *Igκ* V(D)J rearrangement at the pre-B cell stage. At the *Igh* locus, 84% of reproducible ES cell peaks overlapped with pro-B cell peaks, and 97% of neuron peaks overlapped with pro-B cell peaks (Fig. 9B). The high level of overlap of CTCF binding profiles in each cell type strongly contrasts with the distinct histone modification profiles, likely due to the function of distinct transcription factors in each cell type.

## 3 Discussion

**Figure 10:**
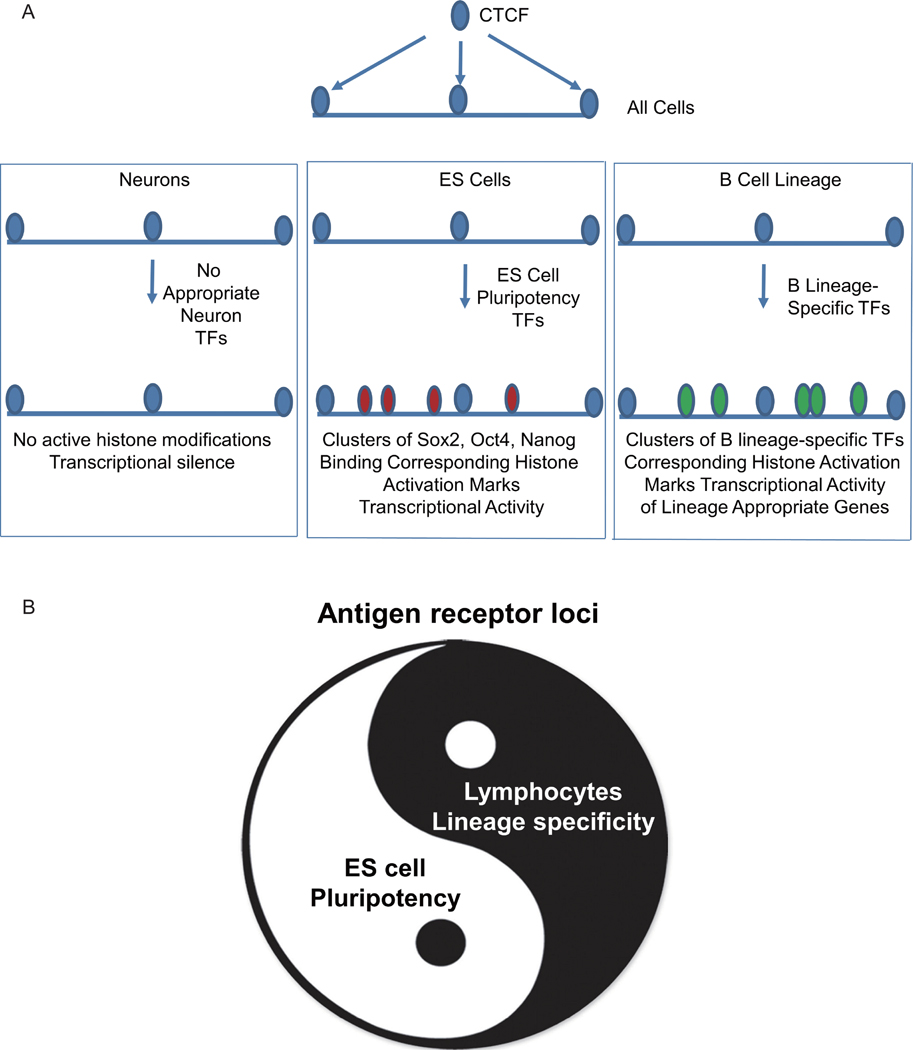
Proposed model. (A) CTCF binds to similar locations in the AgR loci in pro-B cells, ES cells, and neurons. In neurons, we propose lineage-specific transcription factors do not bind to the loci and therefore there are few activating histone marks, and reduced specific transcriptional activity (left panel). In ES cells, Sox 2, Oct 4, and Nanog bind to the loci yielding corresponding histone activating modifications, and transcriptional activation (middle panel). In the B cell lineage, distinct lineage-specific transcription factors bind to different locations resulting in a different pattern of activating histone modifications, and activation of distinct genes (right panel). (B) Epigenetic plasticity depending upon cell type indicative of ES cell pluripotency, in contrast to lymphocyte lineage specificity.

AgR loci are active in B cells and T cells, and we observed active histone modifications and transcription markers at BCR loci in pro-B cells, and TCR loci in mature CD4^+^ T cells. Antigen receptor rearrangement does not occur in ES cells and neurons, and we therefore anticipated the BCR and TCR loci would show epigenetic features of inactivity. Surprisingly, BCR and TCR loci displayed active histone modifications (H3K4me1, H3K4me2, H3K9ac, and H3K27ac) and transcription markers (RNA polymerase II, H3K36me3, and RNA) in ES cells, but not in neurons. ES cells are pluripotent, and have the ability to differentiate into essentially all somatic cells under appropriate conditions [71]. The active epigenetic marks in ES cells may enable ES cells to maintain the capacity to differentiate into lymphocytes until later in development. Terminal differentiation into other lineages, neurons for example, would result in complete silencing of the BCR and TCR loci as the cells are committed to a distinct lineage where BCR and TCR loci are inactive (Fig. 10A).

The mechanism of maintaining the open conformation in ES cells may be due, at least in part, to ES cell-specific transcription factors, Sox2, Oct4 and Nanog, which are essential for the self-renewal and pluripotency of ES cells [72, 73]. We found prominent peaks of Sox2, Oct4, and Nanog binding at the AgR loci in ES cells, which is correlated with ES cell-specific epigenetic features that might maintain openness and enable later lineage-specific epigenetic features. These later lineage-specific events would likely be controlled by transcription factors specific for B and T lymphocytes. Binding by B lineage-specific transcription factors may result in distinct histone modification profiles and enable ubiquitous factors such as YY1 to impact formation of lineage-specific DNA structures such as DNA loops needed for proper gene regulation [69].

Knockout of YY1 has a dramatic impact on chromosomal contraction of *Ig* loci needed for V(D)J rearrangement, DNA loops needed for *Ig* class switch recombination, as well as DNA loops involved in cytokine gene expression in T cells [49, 50, 53, 57, 69, 74–77]. The distinct YY1 binding patterns in ES and pro-B cells suggest that distinct YY1-mediated long-distance DNA interactions may occur in ES compared to pro-B cells

An intriguing possibility is that the open conformation of the AgR loci in ES cells has purposes beyond maintaining an open conformation needed for later lymphocyte development, but instead has some other biological function. Some RNA transcript peaks in ES cells corresponded to V genes in the BCR and TCR loci. If these V genes transcripts are translated they may encode possible receptor-like function in ES cells, though whether functional V gene proteins are synthesized is unknown. Do transcripts from these V gene segments function in unknown developmental mechanisms? Alternatively, are these transcripts non-coding RNAs playing another role in the mechanism of development?

For the *Ig* loci, our studies focused on the pro-B cell stage of development when both *Igκ* and *Igh* loci are undergoing stage-specific locus restructuring. However, only the *Igh* locus is carrying out V(D)J rearrangement at this stage. Consistent with this activity, the *Igh* locus revealed a more open conformation as shown by active histone modifications, and higher RNA transcripts compared to the *Igκ* locus. Similar results were obtained by Loguercio et al. [70] who showed increased *Igκ* openness in pre-B cells compared to pro-B cells. Most of our TCR data are taken from mature CD4^+^ T cells due to the small amount of ChIP-Seq data from DP T cells. Despite the fact that TCR rearrangement has already occurred in mature CD4^+^ T cells, the TCR loci maintain a strong presence of active histone modifications, likely due to the transcriptional activity of the loci to express TCR receptor genes.

A caveat of our study was that ChIP-Seq and RNA-Seq data were obtained from multiple laboratories. In order to minimize that effect, we analyzed multiple histone modification markers (H3K4me1, H3K4me2, H3K9ac, and H3K27ac) and transcriptional markers (RNA polymerase II, H3K36me3, and RNA transcripts) at two BCR loci and three TCR loci. Consistent results were obtained with the multiple markers (histone modifications and transcription) and multiple loci, thus supporting our conclusions. In addition, the high level of overlap in CTCF binding sites at the BCR loci in pro-B cells, ES cells, and neurons provides a positive control for the divergence in histone modifications at these loci we observed in the same cells. Moreover, in many cases the *β-actin* locus showed similar patterns in ES cell, pro-B cells, DP-T cells, CD4^+^ T cells, and neurons.

Our analyses indicate three epigenetic states in distinct cell types. CTCF binding data reveal binding patterns that are common in neurons, ES cells, and pro-B cells (Fig. 10A, top panel). In neurons, there is likely little binding by lineage specific transcription factors at the AgR loci, and thus they contain very few active histone modifications, and low transcriptional activity, (Fig. 10A, left panel). In ES cells, Oct4, Sox2, and Nanog bind to the loci and induce histone modifications resulting in active transcription (Fig. 10A, middle panel). A similar mechanism occurs in pro-B cells, except that B lineage-specific transcription factors likely bind to distinct locations in the loci setting up active histone modifications leading to expression of AgR genes (Fig. 10A, right panel). Our analyses indicate a surprising plasticity and variety of epigenetic landscapes depending upon cell type, and reveal features presumably indicative of ES cell pluripotency, in contrast to lymphocyte lineage specificity (Fig. 10B).

## 4 Methods

### 4.1 Data collection and processing

We downloaded all deep sequencing data from NCBI GEO datasets (Table S1 in the ESM). Four groups of deep sequencing data were analyzed: (1) Histone modifications: H3K4me1, H3K4me2, H3K9ac, and H3K27ac; (2) markers of active transcription: RNA polymerase II, H3K36me3, and RNA transcript data; (3) transcription factors: Sox2, Oct4, Nanog, YY1, Brg1, and CTCF. Partial ChIP-Seq data were uploaded into the UCSC genome browser via the Cistrome data browser (http://cistrome.org/db/#/). Most ChIP-Seq reads were aligned to the mouse reference genome (mm10) using Bowtie 1.1.2 [78]. Duplicated reads were removed by Samtools. BigWig files were generated by bamCoverage function (options: --normalizeUsing RPKM --binSize 100) in the deeptools package. ChIP-Seq data were uploaded into the UCSC genome browser. Read numbers that mapped to different loci were retrieved by Samtools, and the number was normalized into reads per million total mapped reads (RPM) for comparison. Peaks were predicted by MACS2 with *q* < 0.05 [79]. Consensus peaks in multiple ChIP-seq data sets were identified by MSPC algorithm from triplicates [80]. Bedtools was used to process bed files, and overlapped peaks were defined as peaks shared more than 10% of the length of the longer peak. RNA-Seq reads were downloaded from NCBI by SRA Toolkit, and processed by Trimmomatic [81] to trim adapters and to remove low-quality reads. Tophat 2.0.11 [82] with default parameters was used to align good-quality reads to the mouse genome (mm10). Alignment files were processed by Samtools [83] and deeptools [84] to obtain the bigwig files. The genome coverage was normalized as RPKM (reads per kilobase million) using deeptools with a bin size as 100 bp. Additional information is listed in Table S1 in the ESM.

### 4.2 Loci, normalization, and shading

Genomic loci used for our current study are listed as below.*Igκ* (Chr6:67,495,636–70,786,754, mm10), *Igh* (Chr12:113,200,000–116,100,000, mm10), *Tcrα/δ* (Chr14:52397967–54254198, mm10), *Tcrβ* (Chr6:4086129-641588371, mm10) and *β-actin* (Chr5:142,891,416–142,920,715, mm10). In order to compare ChIP-Seq and RNA-Seq data from different cells, we used β-actin as a normalization control and peak height normalizer. Blue, yellow and red shading refer to peaks in only ES cells, lymphoctes (pro-B cells, DP T cells, or mature CD4^+^ T cells), or both, respectively.

### 4.3 Cells

ES cells were primary mouse ES cells or cell line V6.5, R1, E14. Pro-B cells were purified from Rag1 or Rag 2 deficient mouse bone marrow, or sorted with markers B220^+^CD19^+^CD43^+^CD25^–^IgM^–^ from WT bone marrow. DP T cells were purified from thymus with mouse CD4 and CD8 as markers. Mature CD4^+^ T cells were purified from spleen or lymph nodes with mouse CD4 as marker. Information on all tissues and cells are listed in Table S1 in the ESM.

## Supplementary Material

Table S1

Table S2

Table S3

Table S4

Suppl Fig 1 Legend

Figure S1
