# Supplementary material for: Multiple lineage-specific epigenetic landscapes at the antigen receptor loci
Source: Aging Res. Author manuscript; Available in PMC 2024 May 20. (PMC11103674; doi:10.26599/agr.2023.9340010)
Supplement: Table S1 [file NIHMS1983050-supplement-Table_S1.pdf]

Table S1. Public ChIP-Seq, RNA-Seq data cited in the manuscript.

Feature/ Citation	Cell	Strain	Resource	Genotype/variation	GSM number	Coordinates	Coordinates	Coordinates	Coordinates	Coordinates
H3K4me1 [1]	ES cells	129SV/Jae/C57BL/6J	ESC line V6.5	WT	GSM2417088	Chr6:67,495,636-70,786,754 (Igκ, Figure 1A and 6A)	Chr5:142,891,416-142,920,715 (β-actin, Figure 1A and 4)	Chr12:113,200,000-116,100,000 (IgH, Figure 3A and 7A)	Chr14:52397967-54254198 (TCRα/δ, Figure 4)	Chr6:40861296-41588371 (TCRβ, Figure 5)
H3K4me1 [2]	Pro-B cells	C57BL/6	Rag2 deficient pro-B, bone marrow	Rag2$^{-/-}$	GSM932934	Chr6:67,495,636-70,786,754 (Igκ, Figure 1A)	Chr5:142,891,416-142,920,715 (β-actin, Figure 1A)	Chr12:113,200,000-116,100,000 (IgH, Figure 3A)		
H3K4me1 [3]	Neurons	C56BL/6	Neuron, Anterior Cingulate cortex	WT	GSM1939119	Chr6:67,495,636-70,786,754 (Igκ, Figure 1A)	Chr5:142,891,416-142,920,715 (β-actin, Figure 1A and 4)	Chr12:113,200,000-116,100,000 (IgH, Figure 3A)	Chr14:52397967-54254198 (TCRα/δ, Figure 4)	Chr6:40861296-41588371 (TCRβ, Figure 5)
H3K4me2 [1]	ES cells	129SV/Jae/C57BL6J	ESC line V6.5	WT	GSM2417084	Chr6:67,495,636-70,786,754 (Igκ, Figure 1A and 6A)	Chr5:142,891,416-142,920,715 (β-actin, Figure 1A and 4)	Chr12:113,200,000-116,100,000 (IgH, Figure 3A and 7A)	Chr14:52397967-54254198 (TCRα/δ, Figure 4)	Chr6:40861296-41588371 (TCRβ, Figure 5)
H3K4me2 [4]	Pro-B cells	C57BL/6	Rag1 deficient pro-B, bone marrow	Rag1$^{-/-}$	GSM987804	Chr6:67,495,636-70,786,754 (Igκ, Figure 1A)	Chr5:142,891,416-142,920,715 (β-actin, Figure 1A)	Chr12:113,200,000-116,100,000 (IgH, Figure 3A)		
H3K4me2 [5]	Neurons	129Sv-C57BL/6	Terminally differentiated neurons	WT	GSM632054	Chr6:67,495,636-70,786,754 (Igκ, Figure 1A)	Chr5:142,891,416-142,920,715 (β-actin, Figure 1A and 4)	Chr12:113,200,000-116,100,000 (IgH, Figure 3A)	Chr14:52397967-54254198 (TCRα/δ, Figure 4)	Chr6:40861296-41588371 (TCRβ, Figure 5)
H3K9ac [1]	ES cells	129SV/Jae/C57BL/6J	ESC line V6.5	WT	GSM2417092	Chr6:67,495,636-70,786,754 (Igκ, Figure 1A and 6A)	Chr5:142,891,416-142,920,715 (β-actin, Figure 1A and 4)	Chr12:113,200,000-116,100,000 (IgH, Figure 3A and 7A)	Chr14:52397967-54254198 (TCRα/δ, Figure 4)	Chr6:40861296-41588371 (TCRβ, Figure 5)
H3K9ac [6]	Pro-B cells	C57BL/6	Rag2 deficient pro-B, bone marrow	Rag2($^{-/-}$) Ikzf1(fl/+) Cd79a(Cre/+)	GSM1296572	Chr6:67,495,636-70,786,754 (Igκ, Figure 1A)	Chr5:142,891,416-142,920,715 (β-actin, Figure 1A)	Chr12:113,200,000-116,100,000 (IgH, Figure 3A)		
H3K9ac [3]	Neurons	C56BL/6	Neuron, hippocampal CA1	WT	GSM1939075	Chr6:67,495,636-70,786,754 (Igκ, Figure 1A)	Chr5:142,891,416-142,920,715 (β-actin, Figure 1A and 4)	Chr12:113,200,000-116,100,000 (IgH, Figure 3A)	Chr14:52397967-54254198 (TCRα/δ, Figure 4)	Chr6:40861296-41588371 (TCRβ, Figure 5)

H3K27ac [1]	ES cells	129SV/Jae/C57BL/6J	ESC line V6.5	WT	GSM2417096	Chr6:67,495,636-70,786,754 (Igκ, Figure 1A and 6A)	Chr5:142,891,416-142,920,715 (β-actin, Figure 1A and 4)	Chr12:113,200,000-116,100,000 (IgH, Figure 3A and 7A)	Chr14:52397967-54254198 (TCRα/δ, Figure 4)	Chr6:40861296-41588371 (TCRβ, Figure 5)
H3K27ac [7]	Pro-B cells	C57BL/6	Rag2 deficient pro-B, bone marrow	$Rag2^{-/-}$	GSM2255552	Chr6:67,495,636-70,786,754 (Igκ, Figure 1A)	Chr5:142,891,416-142,920,715 (β-actin, Figure 1A)	Chr12:113,200,000-116,100,000 (IgH, Figure 3A)		
H3K27ac [8]	Neurons	129Sv-C57BL/6	Differentiated neurons	WT	GSM1603414	Chr6:67,495,636-70,786,754 (Igκ, Figure 1A)	Chr5:142,891,416-142,920,715 (β-actin, Figure 1A and 4)	Chr12:113,200,000-116,100,000 (IgH, Figure 3A)	Chr14:52397967-54254198 (TCRα/δ, Figure 4)	Chr6:40861296-41588371 (TCRβ, Figure 5)
RNA polymerase II [9]	ES cells	C57BL/6J	Embryonic stem cells	WT	GSM1816100	Chr6:67,495,636-70,786,754 (Igκ, Figure 2)	Chr5:142,891,416-142,920,715 (β-actin, Figure 2 and 4)	Chr12:113,200,000-116,100,000 (IgH, Figure 3B)	Chr14:52397967-54254198 (TCRα/δ, Figure 4)	Chr6:40861296-41588371 (TCRβ, Figure 5)
RNA polymerase II [10]	Pro-B cells	C57BL/6	Rag1 deficient pro-B, bone marrow	$Rag1^{-/-}$	GSM1156660	Chr6:67,495,636-70,786,754 (Igκ, Figure 2)	Chr5:142,891,416-142,920,715 (β-actin, Figure 2)	Chr12:113,200,000-116,100,000 (IgH, Figure 3B)		
RNA polymerase II [5]	Neurons	129Sv-C57BL/6	Terminally differentiated Neurons	WT	GSM632059	Chr6:67,495,636-70,786,754 (Igκ, Figure 2)	Chr5:142,891,416-142,920,715 (β-actin, Figure 2 and 4)	Chr12:113,200,000-116,100,000 (IgH, Figure 3B)	Chr14:52397967-54254198 (TCRα/δ, Figure 4)	Chr6:40861296-41588371 (TCRβ, Figure 5)
H3K36me3 [1]	ES cells	129SV/Jae/C57BL6J	ESC line V6.5	WT	GSM2417108	Chr6:67,495,636-70,786,754 (Igκ, Figure 2)	Chr5:142,891,416-142,920,715 (β-actin, Figure 2)	Chr12:113,200,000-116,100,000 (IgH, Figure 3B)		
H3K36me3 [4]	Pro-B cells	C57BL/6	Rag1 deficient pro-B, bone marrow	$Rag1^{-/-}$	GSM987807	Chr6:67,495,636-70,786,754 (Igκ, Figure 2)	Chr5:142,891,416-142,920,715 (β-actin, Figure 2)	Chr12:113,200,000-116,100,000 (IgH, Figure 3B)		
H3K36me3 [11]	Neurons	C57BL/6	Whole Brain	WT	GSM1000072	Chr6:67,495,636-70,786,754 (Igκ, Figure 2)	Chr5:142,891,416-142,920,715 (β-actin, Figure 2)	Chr12:113,200,000-116,100,000 (IgH, Figure 3B)		
RNA-Seq [12]	ES cells	129/Ola	Embryonic stem cells, total RNA	WT	GSM2537654	Chr6:67,495,636-70,786,754 (Igκ, Figure 2)	Chr5:142,891,416-142,920,715 (β-actin, Figure 2 and 4)	Chr12:113,200,000-116,100,000 (IgH, Figure 3B)	Chr14:52397967-54254198 (TCRα/δ, Figure 4)	Chr6:40861296-41588371 (TCRβ, Figure 5)

RNA-Seq [13]	Pro-B cells	C57BL/6	Rag1 deficient pro-B, bone marrow, total RNA	Rag1$^{-/-}$	GSM1897405	Chr6:67,495,636-70,786,754 (Igκ, Figure 2)	Chr5:142,891,416-142,920,715 (β-actin, Figure 2)	Chr12:113,200,000-116,100,000 (IgH, Figure 3B)		
RNA-Seq [14]	Neurons	ICR	Primary cortical neuron, total RNA	WT	GSM2460426	Chr6:67,495,636-70,786,754 (Igκ, Figure 2)	Chr5:142,891,416-142,920,715 (β-actin, Figure 2 and 4)	Chr12:113,200,000-116,100,000 (IgH, Figure 3B)	Chr14:52397967-54254198 (TCRα/δ, Figure 4)	Chr6:40861296-41588371 (TCRβ, Figure 5)
H3K4me1 [15]	Mature CD4+ T	C57BL/6	T CD4+ cells: CD3+, B220-, CD4+, CD8-, spleen	WT	GSM1441297		Chr5:142,891,416-142,920,715 (β-actin, Figure 4)		Chr14:52397967-54254198 (TCRα/δ, Figure 4)	Chr6:40861296-41588371 (TCRβ, Figure 5)
H3K4me2 [15]	Mature CD4+ T	C57BL/6	T CD4+ cells: CD3+, B220-, CD4+, CD8-, spleen	WT	GSM1441315		Chr5:142,891,416-142,920,715 (β-actin, Figure 4)		Chr14:52397967-54254198 (TCRα/δ, Figure 4)	Chr6:40861296-41588371 (TCRβ, Figure 5)
H3K9ac [16]	Mature CD4+ T	C57BL/6 x 129	TCRbeta high, CD69–, CD24–, CD4+, thymus	WT	GSM1889260		Chr5:142,891,416-142,920,715 (β-actin, Figure 4)		Chr14:52397967-54254198 (TCRα/δ, Figure 4)	Chr6:40861296-41588371 (TCRβ, Figure 5)
H3K9ac [17]	DP T	C57BL/6	CD4+CD8+ (DP) thymocytes, thymus	WT	GSM859501		Chr5:142,891,416-142,920,715 (β-actin, Figure 4)		Chr14:52397967-54254198 (TCRα/δ, Figure 4)	Chr6:40861296-41588371 (TCRβ, Figure 5)
H3K27Ac [18]	Mature CD4+ T	C57BL/6 x 129	Naïve CD4+ T cells, lymph nodes	WT	GSM1694178		Chr5:142,891,416-142,920,715 (β-actin, Figure 4)		Chr14:52397967-54254198 (TCRα/δ, Figure 4)	Chr6:40861296-41588371 (TCRβ, Figure 5)
RNA polymerase II [19]	Mature CD4+ T	C57BL/6	CD4+ T cells, spleen	WT	GSM1903984		Chr5:142,891,416-142,920,715 (β-actin, Figure 4)		Chr14:52397967-54254198 (TCRα/δ, Figure 4)	Chr6:40861296-41588371 (TCRβ, Figure 5)
RNA-Seq [15]	Mature CD4+ T	C57BL/6	CD4+ T cells, B220-, CD3+, CD19-, CD4+, CD8-, spleen, poly A RNA	WT	GSM1464978		Chr5:142,891,416-142,920,715 (β-actin, Figure 4)		Chr14:52397967-54254198 (TCRα/δ, Figure 4)	Chr6:40861296-41588371 (TCRβ, Figure 5)
RNA-Seq [20]	DP T	C56BL/6	CD4+CD8+ (DP) thymocytes, thymus	WT	GSM2634352		Chr5:142,891,416-142,920,715 (β-actin, Figure 4)		Chr14:52397967-54254198 (TCRα/δ, Figure 4)	Chr6:40861296-41588371 (TCRβ, Figure 5)

SOX2 [21]	ES cells	129/Sv	Embryonic stem cells	WT	GSM1910640	Chr6:67,495,636-70,786,754 (Igκ, Figure 6A)		Chr12:113,200,000-116,100,000 (IgH, Figure 7A)		
Oct4 ChIP [21]	ES cells	129/Sv	Embryonic stem cells	WT	GSM1910644	Chr6:67,495,636-70,786,754 (Igκ, Figure 6A)		Chr12:113,200,000-116,100,000 (IgH, Figure 7A)		
Nanog [22]	ES cells	129/Ola	Embryonic stem cells	WT	GSM2123560	Chr6:67,495,636-70,786,754 (Igκ, Figure 6A)		Chr12:113,200,000-116,100,000 (IgH, Figure 7A)		
YY1 [23]	ES cells	129/Ola	BirA-ES cells	WT	GSM788496	Chr6:67,495,636-70,786,754 (Igκ, Figure 8A)		Chr12:113,200,000-116,100,000 (IgH, Figure 8B)		
YY1 [24]	Pro-B cells	C57BL/6	Rag2 deficient pro-B, bone marrow	Yy1(ihCd2/+) Rag2($^{-/-}$) R26(BirA/BirA)	GSM1145864	Chr6:67,495,636-70,786,754 (Igκ, Figure 8A)		Chr12:113,200,000-116,100,000 (IgH, Figure 8B)		
Brg1 [25]	ES cells	129SV	Embryonic stem cells, R1 cell line	WT	GSM2808653	Chr6:67,495,636-70,786,754 (Igκ, Figure 8A)		Chr12:113,200,000-116,100,000 (IgH, Figure 8B)		
Brg1 [26]	Pro-B cells	C57BL/6	Rag1 deficient pro-B, bone marrow	Rag1$^{-/-}$	GSM1635413	Chr6:67,495,636-70,786,754 (Igκ, Figure 8A)		Chr12:113,200,000-116,100,000 (IgH, Figure 8B)		
CTCF [27]	ES cells	Mixed (129-C57BL/6)	Embryonic stem cells	WT	GSM747535	Chr6:67,495,636-70,786,754 (Igκ, Figure 9A)		Chr12:113,200,000-116,100,000 (IgH, Figure 9B)		
CTCF [4]	pro-B cells	C57BL/6	Rag1 deficient pro-B, bone marrow	Rag1$^{-/-}$	GSM987805	Chr6:67,495,636-70,786,754 (Igκ, Figure 9A)		Chr12:113,200,000-116,100,000 (IgH, Figure 9B)		
CTCF [28]	Neurons	129/Ola	Differentiated motor neuron	WT	GSM1468394	Chr6:67,495,636-70,786,754 (Igκ, Figure 9A)		Chr12:113,200,000-116,100,000 (IgH, Figure 9B)		

H3K4me1[29]	ES cells	129SV	Embryonic stem cells, R1 cell line	WT	GSM2808650	Chr6:67,495,636-70,786,754 (Igκ, Table S2)		Chr12:113,200,000-116,100,000 (IgH, Table S3)		
H3K4me1[29]	ES cells	129SV	Embryonic stem cells, R1 cell line	WT	GSM2808660	Chr6:67,495,636-70,786,754 (Igκ, Table S2)		Chr12:113,200,000-116,100,000 (IgH, Table S3)		
H3K4me1[30]	Pro-B cells	C57BL/6	ckit+, B220+, CD19-, CD25-, IgM-, bone marrow	WT	GSM1463434	Chr6:67,495,636-70,786,754 (Igκ, Table S2)		Chr12:113,200,000-116,100,000 (IgH, Table S3)		
H3K4me1[30]	Pro-B cells	C57BL/6	ckit+, B220+, CD19-, CD25-, IgM-, bone marrow	WT	GSM1463435	Chr6:67,495,636-70,786,754 (Igκ, Table S2)		Chr12:113,200,000-116,100,000 (IgH, Table S3)		
H3K4me1[3]	Neurons	C56BL/6	hippocampal CA1	WT	GSM1939116	Chr6:67,495,636-70,786,754 (Igκ, Table S2)		Chr12:113,200,000-116,100,000 (IgH, Table S3)		
H3K4me1[31]	Neurons	B6x129SvJ	Neocortex	WT	GSM2395757	Chr6:67,495,636-70,786,754 (Igκ, Table S2)		Chr12:113,200,000-116,100,000 (IgH, Table S3)		
H3K4me2[29]	ES cells	129SV	Embryonic stem cells, R1 cell line	WT	GSM2808651	Chr6:67,495,636-70,786,754 (Igκ, Table S2)		Chr12:113,200,000-116,100,000 (IgH, Table S3)		
H3K4me2[29]	ES cells	129SV	Embryonic stem cells, R1 cell line	WT	GSM2808674	Chr6:67,495,636-70,786,754 (Igκ, Table S2)		Chr12:113,200,000-116,100,000 (IgH, Table S3)		
H3K4me2[2]	Pro-B cells	C57BL/6	Rag2 deficient pro-B, bone marrow	$Rag2^{-/-}$	GSM932937	Chr6:67,495,636-70,786,754 (Igκ, Table S2)		Chr12:113,200,000-116,100,000 (IgH, Table S3)		
H3K4me2[2]	Pro-B cells	C57BL/6	Rag2 deficient pro-B, bone marrow	$Rag2^{-/-}$	GSM932935	Chr6:67,495,636-70,786,754 (Igκ, Table S2)		Chr12:113,200,000-116,100,000 (IgH, Table S3)		
H3K4me2[32]	Neurons	129SV	Cortical Neurons	WT	GSM1629379	Chr6:67,495,636-70,786,754 (Igκ, Table S2)		Chr12:113,200,000-116,100,000 (IgH, Table S3)		
H3K4me2[33]	Neurons	129Sv-C57Bl/6	Terminally differentiated Neurons	WT	GSM687000	Chr6:67,495,636-70,786,754 (Igκ, Table S2)		Chr12:113,200,000-116,100,000 (IgH, Table S3)		
H3K9ac[34]	ES cells	129P2/Ola	Embryonic stem cells E3.5	WT	GSM1516078	Chr6:67,495,636-70,786,754		Chr12:113,200,000-		

						(Igκ, Table S2)		116,100,000 (IgH, Table S3)		
H3K9ac[11]	ES cells	C57BL/6	mouse embryonic stem cells	WT	GSM1000127	Chr6:67,495,636-70,786,754 (Igκ, Table S2)		Chr12:113,200,000-116,100,000 (IgH, Table S3)		
H3K9ac[35]	Pro-B cells	C57BL/6	Rag2 deficient pro-B, bone marrow	Rag2$^{-/-}$	GSM2055540	Chr6:67,495,636-70,786,754 (Igκ, Table S2)		Chr12:113,200,000-116,100,000 (IgH, Table S3)		
H3K9ac[6]	Pro-B cells	C57BL/6	Rag2 deficient pro-B, bone marrow	Rag2($^{-/-}$) Ikzf1(fl/+) Cd79a(Cre/+)	GSM1296573	Chr6:67,495,636-70,786,754 (Igκ, Table S2)		Chr12:113,200,000-116,100,000 (IgH, Table S3)		
H3K9ac[36]	Neurons	C57BL/6	Differentiated CAD neuron	WT	GSM2039016	Chr6:67,495,636-70,786,754 (Igκ, Table S2)		Chr12:113,200,000-116,100,000 (IgH, Table S3)		
H3K9ac[3]	Neurons	C56BL/6	Hippocampal CA1	WT	GSM1939071	Chr6:67,495,636-70,786,754 (Igκ, Table S2)		Chr12:113,200,000-116,100,000 (IgH, Table S3)		
H3K27ac[37]	ES cells	129/Ola	E14 cell line	WT	GSM2282172	Chr6:67,495,636-70,786,754 (Igκ, Table S2)		Chr12:113,200,000-116,100,000 (IgH, Table S3)		
H3K27ac[37]	ES cells	129/Ola	E14 cell line	WT	GSM2282171	Chr6:67,495,636-70,786,754 (Igκ, Table S2)		Chr12:113,200,000-116,100,000 (IgH, Table S3)		
H3K27ac[38]	Pro-B cells	C57BL/6	CD34+CD19+CD10+, bone marrow	WT	GSM1340626	Chr6:67,495,636-70,786,754 (Igκ, Table S2)		Chr12:113,200,000-116,100,000 (IgH, Table S3)		
H3K27ac[30]	Pro-B cells	C57BL/6	ckit+, B220+, CD19-, CD25-, IgM-, bone marrow	WT	GSM1869131	Chr6:67,495,636-70,786,754 (Igκ, Table S2)		Chr12:113,200,000-116,100,000 (IgH, Table S3)		
H3K27ac[39]	Neurons	129SV	Embryonic stem cells, R1 cell line	WT	GSM2052288	Chr6:67,495,636-70,786,754 (Igκ, Table S2)		Chr12:113,200,000-116,100,000 (IgH, Table S3)		
H3K27ac[8]	Neurons	129-C57BL/6	Day10 Neurons	WT	GSM1603413	Chr6:67,495,636-70,786,754 (Igκ, Table S2)		Chr12:113,200,000-116,100,000 (IgH, Table S3)		
CTCF[27]	ES cells	129-C57BL/6	Embryonic stem cells	WT	GSM747534	Chr6:67,495,636-70,786,754 (Igκ, Table S2)		Chr12:113,200,000-116,100,000 (IgH, Table S3)		

CTCF[40]	ES cells	C57BL/6	Embryonic stem cells	WT	GSM723015	Chr6:67,495,636-70,786,754 (Igκ, Table S2)		Chr12:113,200,000-116,100,000 (IgH, Table S3)			
CTCF[41]	Pro-B cells	C57BL/6	Rag1 deficient pro-B, bone marrow	Rag1$^{-/-}$	GSM1023420	Chr6:67,495,636-70,786,754 (Igκ, Table S2)		Chr12:113,200,000-116,100,000 (IgH, Table S3)			
CTCF[42]	Pro-B cells	C57BL/6	Rag2 deficient pro-B, bone marrow	Rag2$^{-/-}$	GSM1145865	Chr6:67,495,636-70,786,754 (Igκ, Table S2)		Chr12:113,200,000-116,100,000 (IgH, Table S3)			
CTCF[43]	Neurons	C57BL/6	NeuN+ (neuronal), mouse cerebral cortex	WT	GSM2643063	Chr6:67,495,636-70,786,754 (Igκ, Table S2)		Chr12:113,200,000-116,100,000 (IgH, Table S3)			
CTCF[43]	Neurons	C57BL/6	NeuN+ (neuronal), mouse cerebral cortex	WT	GSM2643058	Chr6:67,495,636-70,786,754 (Igκ, Table S2)		Chr12:113,200,000-116,100,000 (IgH, Table S3)			
SOX2 [21]	ES cells	129/Sv	Embryonic stem cells	WT	GSM1910642	Chr6:67,495,636-70,786,754 (Igκ, Table S2)		Chr12:113,200,000-116,100,000 (IgH, Table S3)			
SOX2[44]	ES cells	C57BL/6 -129/Sv	KH2 mouse embryonic stem cells	WT	GSM1842763	Chr6:67,495,636-70,786,754 (Igκ, Table S2)		Chr12:113,200,000-116,100,000 (IgH, Table S3)			
SOX2[44]	ES cells	C57BL/6 -129/Sv	KH2 mouse embryonic stem cells	WT	GSM1842767	Chr6:67,495,636-70,786,754 (Igκ, Table S2)		Chr12:113,200,000-116,100,000 (IgH, Table S3)			
Oct4 ChIP [21]	ES cells	129/Sv	Embryonic stem cells	WT	GSM1910646	Chr6:67,495,636-70,786,754 (Igκ, Table S2)		Chr12:113,200,000-116,100,000 (IgH, Table S3)			
Oct4 ChIP[45]	ES cells	129SV	Embryonic stem cells, R1 cell line	WT	GSM1355155	Chr6:67,495,636-70,786,754 (Igκ, Table S2)		Chr12:113,200,000-116,100,000 (IgH, Table S3)			
Nanog[1]	ES cells	129SV/Jae/C57BL/6J	ESC line V6.5	WT	GSM2417187	Chr6:67,495,636-70,786,754 (Igκ, Table S2)		Chr12:113,200,000-116,100,000 (IgH, Table S3)			
Nanog[46]	ES cells	129SV	Mouse embryonic stem cells	Sox17.Cre-GFP knock-in	GSM1090230	Chr6:67,495,636-70,786,754 (Igκ, Table S2)		Chr12:113,200,000-116,100,000 (IgH, Table S3)			

Supplementary References

[1] C. Chronis, P. Fiziev, B. Papp, S. Butz, G. Bonora, S. Sabri, J. Ernst, and K. Plath, Cooperative Binding of Transcription Factors Orchestrates Reprogramming. Cell 168 (2017) 442-459 e20.

[2] I.D.R. Revilla, I. Bilic, B. Vilagos, H. Tagoh, A. Ebert, I.M. Tamir, L. Smeenk, J. Trupke, A. Sommer, M. Jaritz, and M. Busslinger, The B-cell identity factor Pax5 regulates distinct transcriptional programmes in early and late B lymphopoiesis. EMBO J 31 (2012) 3130-46.

[3] R. Halder, M. Hennion, R.O. Vidal, O. Shomroni, R.U. Rahman, A. Rajput, T.P. Centeno, F. van Bebber, V. Capece, J.C. Garcia Vizcaino, A.L. Schuetz, S. Burkhardt, E. Benito, M. Navarro Sala, S.B. Javan, C. Haass, B. Schmid, A. Fischer, and S. Bonn, DNA methylation changes in plasticity genes accompany the formation and maintenance of memory. Nat Neurosci 19 (2016) 102-10.

[4] Y.C. Lin, C. Benner, R. Mansson, S. Heinz, K. Miyazaki, M. Miyazaki, V. Chandra, C. Bossen, C.K. Glass, and C. Murre, Global changes in the nuclear positioning of genes and intra- and interdomain genomic interactions that orchestrate B cell fate. Nat Immunol 13 (2012) 1196-204.

[5] V.K. Tiwari, M.B. Stadler, C. Wirbelauer, R. Paro, D. Schubeler, and C. Beisel, A chromatin-modifying function of JNK during stem cell differentiation. Nat Genet 44 (2011) 94-100.

[6] T.A. Schwickert, H. Tagoh, S. Gultekin, A. Dakic, E. Axelsson, M. Minnich, A. Ebert, B. Werner, M. Roth, L. Cimmino, R.A. Dickins, J. Zuber, M. Jaritz, and M. Busslinger, Stage-specific control of early B cell development by the transcription factor Ikaros. Nat Immunol 15 (2014) 283-93.

[7] L. Smeenk, M. Fischer, S. Jurado, M. Jaritz, A. Azaryan, B. Werner, M. Roth, J. Zuber, M. Stanulla, M.L. den Boer, C.G. Mullighan, S. Strehl, and M. Busslinger, Molecular role of the PAX5-ETV6 oncoprotein in promoting B-cell acute lymphoblastic leukemia. Embo j 36 (2017) 718-735.

[8] S. Thakurela, S.K. Sahu, A. Garding, and V.K. Tiwari, Dynamics and function of distal regulatory elements during neurogenesis and neuroplasticity. Genome Res 25 (2015) 1309-24.

[9] D. Langer, I. Martianov, D. Alpern, M. Rhinn, C. Keime, P. Dolle, G. Mengus, and I. Davidson, Essential role of the TFIID subunit TAF4 in murine embryogenesis and embryonic stem cell differentiation. Nat Commun 7 (2016) 11063.

[10] N.M. Choi, S. Loguercio, J. Verma-Gaur, S.C. Degner, A. Torkamani, A.I. Su, E.M. Oltz, M. Artyomov, and A.J. Feeney, Deep sequencing of the murine IgH repertoire reveals complex regulation of nonrandom V gene rearrangement frequencies. J Immunol 191 (2013) 2393-402.

[11] F. Yue, Y. Cheng, A. Breschi, J. Vierstra, W. Wu, T. Ryba, R. Sandstrom, Z. Ma, C. Davis, B.D. Pope, Y. Shen, D.D. Pervouchine, S. Djebali, R.E. Thurman, R. Kaul, E. Rynes, A. Kirilusha, G.K. Marinov, B.A. Williams, D. Trout, H. Amrhein, K. Fisher-Aylor, I. Antoshechkin, G. DeSalvo, L.H. See, M. Fastuca, J. Drenkow, C. Zaleski, A. Dobin, P. Prieto, J. Lagarde, G. Bussotti, A. Tanzer, O. Denas, K. Li, M.A. Bender, M. Zhang, R. Byron, M.T. Groudine, D. McCleary, L. Pham, Z. Ye, S. Kuan, L. Edsall, Y.C. Wu, M.D. Rasmussen, M.S. Bansal, M. Kellis, C.A. Keller, C.S. Morrissey, T. Mishra, D. Jain, N. Dogan, R.S. Harris, P. Cayting, T. Kawli, A.P. Boyle, G. Euskirchen, A. Kundaje, S. Lin, Y. Lin, C. Jansen, V.S. Malladi, M.S. Cline, D.T. Erickson, V.M. Kirkup, K. Learned, C.A. Sloan, K.R. Rosenbloom, B. Lacerda de Sousa, K. Beal, M. Pignatelli, P. Flicek, J. Lian, T. Kahveci, D. Lee, W.J. Kent, M. Ramalho Santos, J. Herrero, C. Notredame, A. Johnson, S. Vong, K. Lee, D. Bates, F. Neri, M. Diegel, T. Canfield, P.J. Sabo, M.S. Wilken, T.A. Reh, E. Giste, A. Shafer, T. Kutyavin, E. Haugen, D. Dunn, A.P. Reynolds, S. Neph, R. Humbert, R.S. Hansen, M. De Bruijn, et al., A comparative encyclopedia of DNA elements in the mouse genome. Nature 515 (2014) 355-64.

[12] Y. Semba, A. Harada, K. Maehara, S. Oki, C. Meno, J. Ueda, K. Yamagata, A. Suzuki, M. Onimaru, J. Nogami, S. Okada, K. Akashi, and Y. Ohkawa, Chd2 regulates chromatin for proper gene expression toward differentiation in mouse embryonic stem cells. Nucleic Acids Res 45 (2017) 8758-8772.
[13] E. Kleiman, H. Jia, S. Loguercio, A.I. Su, and A.J. Feeney, YY1 plays an essential role at all stages of B-cell differentiation. Proc Natl Acad Sci U S A 113 (2016) E3911-20.
[14] X. Ding, S. Liu, M. Tian, W. Zhang, T. Zhu, D. Li, J. Wu, H. Deng, Y. Jia, W. Xie, H. Xie, and J.S. Guan, Activity-induced histone modifications govern Neurexin-1 mRNA splicing and memory preservation. Nat Neurosci 20 (2017) 690-699.
[15] D. Lara-Astiaso, A. Weiner, E. Lorenzo-Vivas, I. Zaretsky, D.A. Jaitin, E. David, H. Keren-Shaul, A. Mildner, D. Winter, S. Jung, N. Friedman, and I. Amit, Immunogenetics. Chromatin state dynamics during blood formation. Science 345 (2014) 943-9.
[16] S. Xing, F. Li, Z. Zeng, Y. Zhao, S. Yu, Q. Shan, Y. Li, F.C. Phillips, P.K. Maina, H.H. Qi, C. Liu, J. Zhu, R.M. Pope, C.A. Musselman, C. Zeng, W. Peng, and H.H. Xue, Tcf1 and Lef1 transcription factors establish CD8(+) T cell identity through intrinsic HDAC activity. Nature Immunol. 17 (2016) 695-703.
[17] P. Ntziachristos, A. Tsirigos, P. Van Vlierberghe, J. Nedjic, T. Trimarchi, M.S. Flaherty, D. Ferres-Marco, V. da Ros, Z. Tang, J. Siegle, P. Asp, M. Hadler, I. Rigo, K. De Keersmaecker, J. Patel, T. Huynh, F. Utro, S. Poglio, J.B. Samon, E. Paietta, J. Racevskis, J.M. Rowe, R. Rabadan, R.L. Levine, S. Brown, F. Pflumio, M. Dominguez, A. Ferrando, and I. Aifantis, Genetic inactivation of the polycomb repressive complex 2 in T cell acute lymphoblastic leukemia. Nature Med. 18 (2012) 298-301.
[18] K. Placek, G. Hu, K. Cui, D. Zhang, Y. Ding, J.E. Lee, Y. Jang, C. Wang, J.E. Konkel, J. Song, C. Liu, K. Ge, W. Chen, and K. Zhao, MLL4 prepares the enhancer landscape for Foxp3 induction via chromatin looping. Nature Immunol. 18 (2017) 1035-1045.
[19] A. Onodera, D.J. Tumes, Y. Watanabe, K. Hirahara, A. Kaneda, F. Sugiyama, Y. Suzuki, and T. Nakayama, Spatial Interplay between Polycomb and Trithorax Complexes Controls Transcriptional Activity in T Lymphocytes. Mol Cell Biol 35 (2015) 3841-53.
[20] J.L. Johnson, G. Georgakilas, J. Petrovic, M. Kurachi, S. Cai, C. Harly, W.S. Pear, A. Bhandoola, E.J. Wherry, and G. Vahedi, Lineage-Determining Transcription Factor TCF-1 Initiates the Epigenetic Identity of T Cells. Immunity 48 (2018) 243-257.e10.
[21] Z. Liu, and W.L. Kraus, Catalytic-Independent Functions of PARP-1 Determine Sox2 Pioneer Activity at Intractable Genomic Loci. Mol Cell 65 (2017) 589-603.e9.
[22] T.J. Stevens, D. Lando, S. Basu, L.P. Atkinson, Y. Cao, S.F. Lee, M. Leeb, K.J. Wohlfahrt, W. Boucher, A. O'Shaughnessy-Kirwan, J. Cramard, A.J. Faure, M. Ralser, E. Blanco, L. Morey, M. Sanso, M.G.S. Palayret, B. Lehner, L. Di Croce, A. Wutz, B. Hendrich, D. Klenerman, and E.D. Laue, 3D structures of individual mammalian genomes studied by single-cell Hi-C. Nature 544 (2017) 59-64.
[23] P. Vella, I. Barozzi, A. Cuomo, T. Bonaldi, and D. Pasini, Yin Yang 1 extends the Myc-related transcription factors network in embryonic stem cells. Nucleic Acids Res 40 (2012) 3403-18.
[24] J. Medvedovic, A. Ebert, H. Tagoh, I.M. Tamir, T.A. Schwickert, M. Novatchkova, Q. Sun, P.J.H.i.t. Veld, C. Guo, H.S. Yoon, Y. Denizot, S.J.B. Holwerda, W. de Laat, M. Cogné, Y. Shi, F.W. Alt, and M. Busslinger, Flexible Long-Range Loops in the VH Gene Region of the Igh Locus Facilitate the Generation of a Diverse Antibody Repertoire. Immunity 39 (2013) 229-244.
[25] A. Local, H. Huang, C.P. Albuquerque, N. Singh, A.Y. Lee, W. Wang, C. Wang, J.E. Hsia, A.K. Shiau, K. Ge, K.D. Corbett, D. Wang, H. Zhou, and B. Ren, Identification of H3K4me1-associated proteins at mammalian enhancers. Nature Genet. 50 (2018) 73-82.
[26] C. Bossen, C.S. Murre, A.N. Chang, R. Mansson, H.R. Rodewald, and C. Murre, The chromatin remodeler Brg1 activates enhancer repertoires to establish B cell identity and modulate cell growth. Nature Immunol. 16 (2015) 775-84.

[27] M.B. Stadler, R. Murr, L. Burger, R. Ivanek, F. Lienert, A. Scholer, E. van Nimwegen, C. Wirbelauer, E.J. Oakeley, D. Gaidatzis, V.K. Tiwari, and D. Schubeler, DNA-binding factors shape the mouse methylome at distal regulatory regions. Nature 480 (2011) 490-5.
[28] V. Narendra, P.P. Rocha, D. An, R. Raviram, J.A. Skok, E.O. Mazzoni, and D. Reinberg, CTCF establishes discrete functional chromatin domains at the Hox clusters during differentiation. Science 347 (2015) 1017-21.
[29] A. Local, H. Huang, C.P. Albuquerque, N. Singh, A.Y. Lee, W. Wang, C. Wang, J.E. Hsia, A.K. Shiau, K. Ge, K.D. Corbett, D. Wang, H. Zhou, and B. Ren, Identification of H3K4me1-associated proteins at mammalian enhancers. Nat Genet 50 (2018) 73-82.
[30] M.A. Choukrallah, S. Song, A.G. Rolink, L. Burger, and P. Matthias, Enhancer repertoires are reshaped independently of early priming and heterochromatin dynamics during B cell differentiation. Nat Commun 6 (2015) 8324.
[31] R.M. Walsh, E.Y. Shen, R.C. Bagot, A. Anselmo, Y. Jiang, B. Javidfar, G.J. Wojtkiewicz, J. Cloutier, J.W. Chen, R. Sadreyev, E.J. Nestler, S. Akbarian, and K. Hochedlinger, Phf8 loss confers resistance to depression-like and anxiety-like behaviors in mice. Nat Commun 8 (2017) 15142.
[32] F. Telese, Q. Ma, P.M. Perez, D. Notani, S. Oh, W. Li, D. Comoletti, K.A. Ohgi, H. Taylor, and M.G. Rosenfeld, LRP8-Reelin-Regulated Neuronal Enhancer Signature Underlying Learning and Memory Formation. Neuron 86 (2015) 696-710.
[33] F. Lienert, F. Mohn, V.K. Tiwari, T. Baubec, T.C. Roloff, D. Gaidatzis, M.B. Stadler, and D. Schubeler, Genomic prevalence of heterochromatic H3K9me2 and transcription do not discriminate pluripotent from terminally differentiated cells. PLoS Genet 7 (2011) e1002090.
[34] J. Molitor, J.P. Mallm, K. Rippe, and F. Erdel, Retrieving Chromatin Patterns from Deep Sequencing Data Using Correlation Functions. Biophys J 112 (2017) 473-490.
[35] A. Pasquarella, A. Ebert, G. Pereira de Almeida, M. Hinterberger, M. Kazerani, A. Nuber, J. Ellwart, L. Klein, M. Busslinger, and G. Schotta, Retrotransposon derepression leads to activation of the unfolded protein response and apoptosis in pro-B cells. Development 143 (2016) 1788-99.
[36] P. Mews, G. Donahue, A.M. Drake, V. Luczak, T. Abel, and S.L. Berger, Acetyl-CoA synthetase regulates histone acetylation and hippocampal memory. Nature 546 (2017) 381-386.
[37] A.H. Juan, S. Wang, K.D. Ko, H. Zare, P.F. Tsai, X. Feng, K.O. Vivanco, A.M. Ascoli, G. Gutierrez-Cruz, J. Krebs, S. Sidoli, A.L. Knight, R.A. Pedersen, B.A. Garcia, R. Casellas, J. Zou, and V. Sartorelli, Roles of H3K27me2 and H3K27me3 Examined during Fate Specification of Embryonic Stem Cells. Cell Rep 17 (2016) 1369-1382.
[38] A.A. Lane, B. Chapuy, C.Y. Lin, T. Tivey, H. Li, E.C. Townsend, D. van Bodegom, T.A. Day, S.C. Wu, H. Liu, A. Yoda, G. Alexe, A.C. Schinzel, T.J. Sullivan, S. Malinge, J.E. Taylor, K. Stegmaier, J.D. Jaffe, M. Bustin, G. te Kronnie, S. Izraeli, M.H. Harris, K.E. Stevenson, D. Neuberg, L.B. Silverman, S.E. Sallan, J.E. Bradner, W.C. Hahn, J.D. Crispino, D. Pellman, and D.M. Weinstock, Triplication of a 21q22 region contributes to B cell transformation through HMGN1 overexpression and loss of histone H3 Lys27 trimethylation. Nat Genet 46 (2014) 618-23.
[39] W. Zhang, W. Xia, Q. Wang, A.J. Towers, J. Chen, R. Gao, Y. Zhang, C.A. Yen, A.Y. Lee, Y. Li, C. Zhou, K. Liu, J. Zhang, T.P. Gu, X. Chen, Z. Chang, D. Leung, S. Gao, Y.H. Jiang, and W. Xie, Isoform Switch of TET1 Regulates DNA Demethylation and Mouse Development. Mol Cell 64 (2016) 1062-1073.
[40] Y. Shen, F. Yue, D.F. McCleary, Z. Ye, L. Edsall, S. Kuan, U. Wagner, J. Dixon, L. Lee, V.V. Lobanenkov, and B. Ren, A map of the cis-regulatory sequences in the mouse genome. Nature 488 (2012) 116-20.

[41] H.Y. Shih, J. Verma-Gaur, A. Torkamani, A.J. Feeney, N. Galjart, and M.S. Krangel, Tcra gene recombination is supported by a Tcra enhancer- and CTCF-dependent chromatin hub. Proc Natl Acad Sci U S A 109 (2012) E3493-502.
[42] J. Medvedovic, A. Ebert, H. Tagoh, I.M. Tamir, T.A. Schwickert, M. Novatchkova, Q. Sun, P.J. Huis In 't Veld, C. Guo, H.S. Yoon, Y. Denizot, S.J. Holwerda, W. de Laat, M. Cogne, Y. Shi, F.W. Alt, and M. Busslinger, Flexible long-range loops in the VH gene region of the Igh locus facilitate the generation of a diverse antibody repertoire. Immunity 39 (2013) 229-44.
[43] Y. Jiang, Y.E. Loh, P. Rajarajan, T. Hirayama, W. Liao, B.S. Kassim, B. Javidfar, B.J. Hartley, L. Kleofas, R.B. Park, B. Labonte, S.M. Ho, S. Chandrasekaran, C. Do, B.R. Ramirez, C.J. Peter, W.J. C, B.M. Safaie, H. Morishita, P. Roussos, E.J. Nestler, A. Schaefer, B. Tycko, K.J. Brennand, T. Yagi, L. Shen, and S. Akbarian, The methyltransferase SETDB1 regulates a large neuron-specific topological chromatin domain. Nat Genet 49 (2017) 1239-1250.
[44] C. Galonska, M.J. Ziller, R. Karnik, and A. Meissner, Ground State Conditions Induce Rapid Reorganization of Core Pluripotency Factor Binding before Global Epigenetic Reprogramming. Cell Stem Cell 17 (2015) 462-70.
[45] C. Buecker, R. Srinivasan, Z. Wu, E. Calo, D. Acampora, T. Faial, A. Simeone, M. Tan, T. Swigut, and J. Wysocka, Reorganization of enhancer patterns in transition from naive to primed pluripotency. Cell Stem Cell 14 (2014) 838-53.
[46] A.E. Kartikasari, J.X. Zhou, M.S. Kanji, D.N. Chan, A. Sinha, A. Grapin-Botton, M.A. Magnuson, W.E. Lowry, and A. Bhushan, The histone demethylase Jmjd3 sequentially associates with the transcription factors Tbx3 and Eomes to drive endoderm differentiation. EMBO J 32 (2013) 1393-408.
