# Supplementary material for: Multiple lineage-specific epigenetic landscapes at the antigen receptor loci
Source: Aging Res. Author manuscript; Available in PMC 2024 May 20. (PMC11103674; doi:10.26599/agr.2023.9340010)
Supplement: Table S2 [file NIHMS1983050-supplement-Table_S2.pdf]

Table S2. Differential number of peaks between ES cells, pro-B cells and neurons at the *Igκ* locus.

H3K4me1

ES cells		Pro-B cells		Neuron	
GEO	Peak number	GEO	Peak number	GEO	Peak number
GSM2417088	53	GSM932934	71	GSM1939119	1
GSM2808650	29	GSM1463434	264	GSM1939116	3
GSM2808660	27	GSM1463435	130	GSM2395757	2

H3K4me2

ES cells		Pro-B cells		Neurons	
GEO	Peak number	GEO	Peak number	GEO	Peak number
GSM2417084	88	GSM987804	116	GSM632054	9
GSM2808651	46	GSM932937	33	GSM1629379	4
GSM2808674	45	GSM932935	42	GSM687000	9

H3K9ac

ES cells		Pro-B cells		Neurons	
GEO	Peak number	GEO	Peak number	GEO	Peak number
GSM2417092	40	GSM1296572	20	GSM1939075	1
GSM1516078	5	GSM2055540	87	GSM2039016	12
GSM1000127	10	GSM1296573	17	GSM1939071	1

H3K27ac

ES cells		Pro-B cells		Neurons	
GEO	Peak number	GEO	Peak number	GEO	Peak number
GSM2417096	64	GSM2255552	91	GSM1603414	0
GSM2282172	55	GSM1340626	168	GSM2052288	2
GSM2282171	56	GSM1869131	146	GSM1603413	2

CTCF

ES cells		Pro-B cells		Neuron	
GEO	Peak number	GEO	Peak number	GEO	Peak number
GSM747534	84	GSM987805	59	GSM2643063	39
GSM723015	40	GSM1023420	28	GSM2643058	51
GSM747535	89	GSM1145865	46	GSM1468394	43

ES cells

SOX2		Oct4		Nanog	
GEO	Peak number	GEO	Peak number	GEO	Peak number
GSM1910642	11	GSM1910644	32	GSM2123560	25
GSM1842763	20	GSM1910646	38	GSM2417187	23
GSM1842767	21	GSM1355155	25	GSM1090230	15
