# Supplementary material for: Multiple lineage-specific epigenetic landscapes at the antigen receptor loci
Source: Aging Res. Author manuscript; Available in PMC 2024 May 20. (PMC11103674; doi:10.26599/agr.2023.9340010)
Supplement: Table S3 [file NIHMS1983050-supplement-Table_S3.pdf]

Table S3. Differential number of peaks between ES cells, pro-B cells and neurons at the *IgH* locus.

H3K4me1

ES cells		Pro-B cells		Neuron	
GEO	Peak number	GEO	Peak number	GEO	Peak number
GSM2417088	13	GSM932934	232	GSM1939119	0
GSM2808650	15	GSM1463434	245	GSM1939116	2
GSM2808660	16	GSM1463435	217	GSM2395757	1

H3K4me2

ES cells		Pro-B cells		Neurons	
GEO	Peak number	GEO	Peak number	GEO	Peak number
GSM2417084	44	GSM987804	97	GSM632054	3
GSM2808651	19	GSM932937	100	GSM1629379	5
GSM2808674	23	GSM932935	108	GSM687000	3

H3K9ac

ES cells		Pro-B cells		Neurons	
GEO	Peak number	GEO	Peak number	GEO	Peak number
GSM2417092	34	GSM1296572	96	GSM1939075	5
GSM1516078	43	GSM2055540	193	GSM2039016	16
GSM1000127	32	GSM1296573	93	GSM1939071	2

H3K27ac

ES cells		Pro-B cells		Neurons	
GEO	Peak number	GEO	Peak number	GEO	Peak number
GSM2417096	20	GSM2255552	208	GSM1603414	3
GSM2282172	27	GSM1340626	126	GSM2052288	4
GSM2282171	23	GSM1869131	136	GSM1603413	4

CTCF

ES cells		Pro-B cells		Neuron	
GEO	Peak number	GEO	Peak number	GEO	Peak number
GSM747534	130	GSM987805	118	GSM2643063	39
GSM723015	69	GSM1023420	141	GSM2643058	49
GSM747535	145	GSM1145865	156	GSM1468394	33

ES cells

SOX2		Oct4		Nanog	
GEO	Peak number	GEO	Peak number	GEO	Peak number
GSM1910642	6	GSM1910644	15	GSM2123560	10
GSM1842763	12	GSM1910646	14	GSM2417187	9
GSM1842767	9	GSM1355155	7	GSM1090230	17
