# Supplementary material for: Multiple lineage-specific epigenetic landscapes at the antigen receptor loci
Source: Aging Res. Author manuscript; Available in PMC 2024 May 20. (PMC11103674; doi:10.26599/agr.2023.9340010)
Supplement: Table S4 [file NIHMS1983050-supplement-Table_S4.pdf]

Table S3. Normalized trancript reads at Igk and IgH loci in ES cells, pro-B cells, and neuro[illegible]

		Igk	IgH
**RNA-Seq**	**ES**	245	204
	**Pro-B**	587	2183
	**Neuron**	75	103

ns.
