# Supplementary material for: Multiple lineage-specific epigenetic landscapes at the antigen receptor loci
Source: Aging Res. Author manuscript; Available in PMC 2024 May 20. (PMC11103674; doi:10.26599/agr.2023.9340010)
Supplement: Suppl Fig 1 Legend [file NIHMS1983050-supplement-Suppl_Fig_1_Legend.pdf]

Figure S1. Schematic representation of lymphocyte development. ChIP-seq data from pro-B, DP T cells, and mature CD4+ T cells (highlighted in red, green and blue, respectively) were used for this manuscript.
