# Supplementary figures and images for: Multiple lineage-specific epigenetic landscapes at the antigen receptor loci
Source: Aging Res. Author manuscript; Available in PMC 2024 May 20. (PMC11103674; doi:10.26599/agr.2023.9340010)

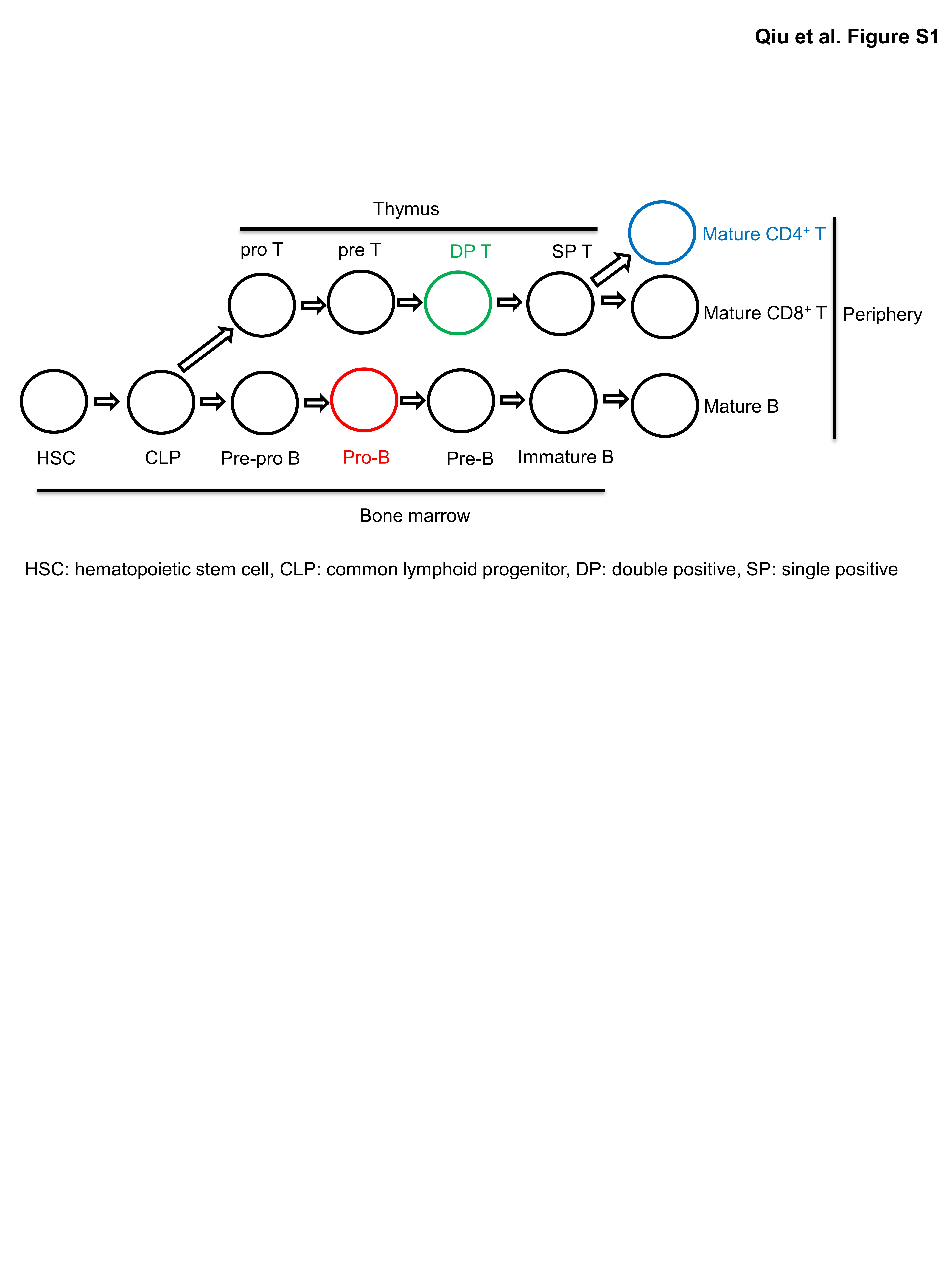

Supplement: Figure S1 [file NIHMS1983050-supplement-Figure_S1.jpg]
